# Diabetes mellitus, prediabetes and the risk of Parkinson’s disease: a systematic review and meta-analysis of 15 cohort studies with 29.9 million participants and 86,345 cases

**DOI:** 10.1007/s10654-023-00970-0

**Published:** 2023-04-25

**Authors:** Dagfinn Aune, Sabrina Schlesinger, Yahya Mahamat-Saleh, Bang Zheng, Chinedu T. Udeh-Momoh, Lefkos T. Middleton

**Affiliations:** 1 Department of Epidemiology and Biostatistics, School of Public Health, Imperial College London, St. Mary’s Campus, Norfolk Place, W2 1PG Paddington, London UK; 2 Department of Nutrition, Oslo New University College, Oslo, Norway; 3 Department of Endocrinology, Morbid Obesity and Preventive Medicine, Oslo University Hospital, Oslo, Norway; 4 Institute for Biometry and Epidemiology, German Diabetes Center, Leibniz Institute for Diabetes Research at the Heinrich-Heine-University Düsseldorf, Düsseldorf, Germany; 5 German Center for Diabetes Research (DZD), Munich-Neuherberg, Germany; 6 International Agency for Research on Cancer, Lyon, France; 7 Ageing Epidemiology Research Unit, School of Public Health, Imperial College London, London, UK; 8 Public Health Directorate, Imperial College NHS Healthcare Trust, London, UK

**Keywords:** Diabetes mellitus, Parkinson’s disease, Systematic review, Meta-analysis

## Abstract

A diagnosis of diabetes mellitus and prediabetes has been associated with increased risk of Parkinson’s disease (PD) in several studies, but results have not been entirely consistent. We conducted a systematic review and meta-analysis of cohort studies on diabetes mellitus, prediabetes and the risk of PD to provide an up-to-date assessment of the evidence. PubMed and Embase databases were searched for relevant studies up to 6th of February 2022. Cohort studies reporting adjusted relative risk (RR) estimates and 95% confidence intervals (CIs) for the association between diabetes, prediabetes and Parkinson’s disease were included. Summary RRs (95% CIs) were calculated using a random effects model. Fifteen cohort studies (29.9 million participants, 86,345 cases) were included in the meta-analysis. The summary RR (95% CI) of PD for persons with diabetes compared to persons without diabetes was 1.27 (1.20–1.35, I^2^ = 82%). There was no indication of publication bias, based on Egger’s test (p = 0.41), Begg’s test (p = 0.99), and inspection of the funnel plot. The association was consistent across geographic regions, by sex, and across several other subgroup and sensitivity analyses. There was some suggestion of a stronger association for diabetes patients reporting diabetes complications than for diabetes patients without complications (RR = 1.54, 1.32–1.80 [n = 3] vs. 1.26, 1.16–1.38 [n = 3]), vs. those without diabetes (p_heterogeneity_=0.18). The summary RR for prediabetes was 1.04 (95% CI: 1.02–1.07, I^2^ = 0%, n = 2). Our results suggest that patients with diabetes have a 27% increased relative risk of developing PD compared to persons without diabetes, and persons with prediabetes have a 4% increase in RR compared to persons with normal blood glucose. Further studies are warranted to clarify the specific role age of onset or duration of diabetes, diabetic complications, glycaemic level and its long-term variability and management may play in relation to PD risk.

**Supplementary information:**

The online version contains supplementary material available at 10.1007/s10654-023-00970-0.

## Introduction

The prevalence of diabetes mellitus (DM) has increased to epidemic levels over the last decades, concurrent with the increased rates of overweight and obesity globally. The number of adults with diabetes, approximately 90% of whom have the type 2 DM form (T2D), increased from 108 million in 1980 to 422 million in 2014 globally, and the age-standardized diabetes prevalence increased from 4.3 to 9.0% in men and from 5.0 to 7.9% in women in the same period [1]. The number of diabetes patients globally has further increased to 463 million in 2019 [2]. Persons with diabetes are at increased risk of various complications including retinopathy [3], neuropathy [3] and nephropathy [3], as well as a large number of chronic diseases including cardiovascular diseases [4], several cancers [4], and several other diseases across multiple organ systems [4, 5].

A growing body of evidence suggests that diabetes may also significantly increase the risk for Alzheimer’s disease (AD), Parkinson’s disease (PD), and other progressive late onset neurodegenerative diseases [4, 6], with an ever growing number of sufferers, in parallel to increases in life expectancy [7]. Several epidemiological studies have investigated the association between a history of diabetes mellitus and the risk of PD [8–21]; but the results have not been entirely consistent with some studies reporting a positive association [9–11, 13–17, 19–21], whilst others found no clear association [8, 12, 18]. A previous meta-analysis of seven cohort studies, that included 1,761,632 individuals reported a 38% increased risk of PD among patients with diabetes [22]. An earlier meta-analysis of five cohort studies (reported in four publications and including 2975 PD cases and 483,551 participants) found a similar 37% increase in risk of PD among persons with diabetes [23]. However, results from case-control studies showed a contrasting non-significant reduction in risk [23]. Two more recent meta-analyses including 7 and 10 cohort studies both reported a 29% increase in risk of PD among diabetes patients [24, 25]. Six cohort studies with > 65,000 PD cases and > 20.4 million participants have since been published [13, 16, 17, 19–21], and all of these reported increased risk [13, 16, 17, 19–21]. A few studies also suggested increased PD risk in persons with prediabetes [21, 26] or diabetes complications [15, 16], although results were not always consistent [10]. To further clarify whether there is an association between DM, prediabetes and PD we therefore conducted a systematic review and meta-analysis of cohort studies to provide a more up-to-date and comprehensive assessment of the evidence, and to evaluate potential sources of heterogeneity between studies.

## Materials and methods

### Search strategy

We searched PubMed and Embase databases up to 6th of February 2022 for eligible studies. The search terms used are provided in the supplementary text. We followed the PRISMA criteria for reporting of meta-analyses [27]. The reference lists of the included publications were screened for further potentially relevant studies. DA and SS conducted the screening of the literature search independently.

### Study selection and inclusion criteria

We included published retrospective and prospective cohort studies and case-control studies nested within cohorts that reported adjusted relative risk (RR) estimates (including hazard ratios, risk ratios and odds ratios) and 95% confidence intervals (CIs) for the association between DM and the risk of PD. When several publications were published from the same cohort we included the publication with the largest sample size, with one exception, where the included study adjusted for several additional confounders [14]. A list of the excluded studies and the corresponding rationale can be found in Supplementary Table 1.

### Data extraction

The following data were extracted from each study: The first author’s last name, publication year, country where the study was conducted, the name of the study, study period and duration of follow-up, sample size, sex, age, number of cases, type of diabetes, subgroup, RRs and 95% CIs and variables adjusted for in the analysis. The data extraction was conducted by DA and checked for accuracy by YMS.

### Study quality assessment

Study quality was assessed using a modified version of the Newcastle Ottawa Scale (NOS) which rates studies according to selection, comparability and outcome assessment [28]. The version applied include the following modifications that, in our view, address important limitations of NOS [29] and can potentially add value to the estimated score: (1) the point regarding representativeness was removed, as it is not relevant for study quality; (2) scoring 0.25 point per confounding factor adjusted for, up to a maximum of 2 points, instead for giving 2 points for adjustments for two confounders. This was deemed justifiable, as studies with relatively crude adjustment (e.g. for age and sex) could still receive, in the original scale, a maximum score but could still be prone to confounding; and (3) for the outcome assessment, we allocated half a point for record linkage and one point for independent outcome assessment by another physician or healthcare worker or for validated assessment of the outcome. This modified NOS gave a total score range from 0 to 8, instead of the range of 0 to 9 of the original scale.

### Statistical methods

We used random effects models to calculate summary RRs (95% CIs) for the association between DM and PD risk [30]. The average of the natural logarithm of the RRs was estimated and the RR from each study was weighted using random effects weights [30]. For studies that reported results stratified by sex or diabetes complications, but not overall, we pooled the results from each subgroup using a fixed effects model before including the study in the overall analysis.

Heterogeneity between studies was evaluated using the Q test and I^2^ statistics [31]. I^2^ is a measure of how much of the heterogeneity is due to between study variation rather than chance, ranging from 0 to 100%. We conducted main meta-analyses (all studies combined) and stratified by study characteristics such as sex, duration of follow-up, exclusion of early follow-up, geographic location, number of cases, assessment of Parkinson’s disease diagnosis, type of diabetes, assessment of diabetes, timing of diabetes diagnosis, diabetes duration, presence of diabetes complications (including episodes of hypoglycaemia, retinopathy, peripheral neuropathy and nephropathy), study quality and by adjustment for confounding factors to investigate potential sources of heterogeneity. Furthermore, we calculated E-values for the association between diabetes and PD, to assess the potential impact of unmeasured or uncontrolled confounding [32]. The E-value is defined as the minimum strength that an unmeasured or uncontrolled confounder would have with both the exposure and the outcome to fully explain away the observed association. We used the World Cancer Research Fund criteria for grading the overall evidence regarding DM and PD, with possible gradings rated as convincing, probable, limited-suggestive, limited - no conclusion, or substantial effect on risk unlikely [33]. The criteria are described in more detail in Supplementary Table 2.

Publication bias was assessed using Egger’s test [34] and Begg’s test [35] and by inspection of funnel plots. The statistical analyses were conducted using the software package Stata, version 13.0 (StataCorp, Texas, US).

## Results

**Fig. 1:**
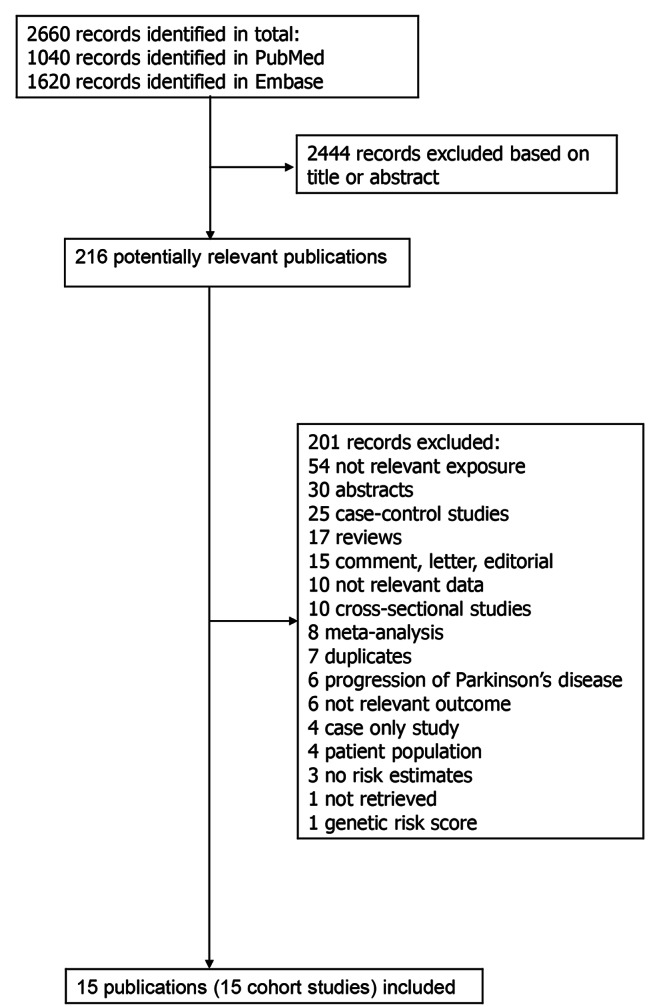
Flow-chart of study selection

A total of 2660 records were screened and of these we identified 15 population-based cohort studies (15 publications, 14 risk estimates) [8–21] with 29.9 million participants and 86,345 PD cases that were included in the meta-analysis (Fig. 1; Table 1). One publication reported results for two cohort studies combined [8]. One additional publication [26] was included in a subgroup analysis by duration of diabetes as it overlapped with another publication with a larger sample, from the same study [16] that was used for the main analysis, however, it [26] was also included in the analysis of prediabetes and PD. Six of the included studies (five publications) were from the United States (US) [8, 10–12, 17], five studies from Europe [9, 13, 15, 20, 21] and four studies from Asia [14, 16, 18, 19] (Table 1). All studies were conducted in adult populations, the age at baseline ranged from 18 to 84 years for the studies that provided an age range. Four studies only used self-reported diabetes [10–12, 19], while eleven studies (10 publications) [8, 9, 13–18, 20, 21] also included either registry linkage, blood glucose, and/or a validation study of diabetes diagnoses. PD diagnoses were validated or assessed independently by health practitioners in six studies (five publications) [8–12], while nine studies [13–21] used a combination of self-report and registry linkage or registry linkage only. The mean (median) study quality scores, using the modified NOS, was 5.9 (5.75) out of 8; corresponding scores for each item across studies are shown in Supplementary Table 3. Lack of reporting on adequacy (completeness) of follow-up was the most common reason for a lower than optimal score. Other relatively common limitations that contributed to a lower than optimal score across multiple studies include using only self-reported data on diabetes, not having an independent or validated outcome assessment and inadequate adjustment for confounders.

**Table 1:** Prospective studies of diabetes mellitus and Parkinson’s disease

First author, publication year, country	Study name or description	Study period	Number of participants, sex, age, number of cases	Type of diabetes, subgroup	Comparison	Relative risk (95% confidence interval)	Adjustment for confounders
Simon KC et al, 2007, USA	Nurses’ Health Study & Health Professionals Follow-up Study	1976–2000, 22.9 years follow-up 1986–2000, 12.6 years follow-up	121046 women and 50833 men, age 30–55 years/40–75 years: 530 cases	Diabetes mellitus	Yes vs. No	1.04 (0.74–1.46)	Age, smoking status, pack-years
Hu G et al, 2007, Finland	Finland MONICA study	1972, 1977, 1982, 1987, 1992, 1997–2002, 18 years follow-up	25168 men and 26384 women, age 26–74 years: 324/309 cases	Type 2 diabetes, all	Yes vs. no	1.83 (1.21–2.76)	Age, sex (all), study year, BMI, SBP, cholesterol, education, leisure-time physical activity, cigarette smoking status and cigarettes per day, coffee, tea, alcohol
				Type 2 diabetes, men	Yes vs. no	1.78 (1.01–3.12)	
				Type 2 diabetes, women	Yes vs. no	1.91 (1.04–3.52)	
				Type 2 diabetes, excluding first 5 years of follow-up	Yes vs. no	1.88 (1.19–2.99)	
Driver JA et al, 2008, USA	Physicians’ Health Study	1982 – NA, 23.1 years follow-up	21841 men, age 40–84 years: 556 cases	Type 2 diabetes, all	Yes vs. no	1.34 (1.01–1.77)	Age, smoking status, alcohol use, BMI, physical activity, hypertension, cholesterol level
				Type 2 diabetes, age < 55 years	Yes vs. no	1.12 (0.61–2.07)	
				Type 2 diabetes, age 55–64	Yes vs. no	1.57 (1.04–2.38)	
				Type 2 diabetes, age ≥ 65	Yes vs. no	1.25 (0.76–2.05)	
				Type 2 diabetes, BMI < 25	Yes vs. no	1.88 (1.28–2.77)	
				Type 2 diabetes, BMI 25-<30	Yes vs. no	1.14 (0.75–1.72)	
				Type 2 diabetes, BMI ≥ 30	Yes vs. no	0.36 (0.08–1.59)	
				Type 2 diabetes, no complications	Yes vs. no	1.63 (1.14–2.33)	
				Type 2 diabetes, complications	Yes vs. no	1.10 (0.74–1.64)	
				Type 2 diabetes, < 5 years duration	Yes vs. no	7.17 (4.59–11.20)	
				Type 2 diabetes, 5–9 years	Yes vs. no	2.03 (1.22–3.36)	
				Type 2 diabetes, 10–14 years	Yes vs. no	0.82 (0.42–1.60)	
				Type 2 diabetes, ≥ 15 years	Yes vs. no	0.73 (0.45–1.18)	
				Type 2 diabetes, < 63.7 years at diabetes onset	Yes vs. no	1.18 (0.78–1.79)	
				Type 2 diabetes, ≥ 63.7 years at diabetes onset	Yes vs. no	1.49 (1.04–2.11)	
Palacios N et al, 2011, USA	Cancer Prevention Study 2 Nutrition Cohort	1992–2005, ~ 11.7 years follow-up	147096 men and women, age 63.6/62.0 years: 656 cases	Diabetes, all	Yes vs. no	0.88 (0.62–1.25)	Age, smoking – pack-years, alcohol, caffeine, calories, dairy intake, pesticide exposure, physical activity, education
				Diabetes, men	Yes vs. no	0.87 (0.57–1.33)	
				Diabetes, women	Yes vs. no	0.90 (0.48–1.66)	
Xu Q et al, 2011, USA	NIH-AARP Diet and Health Study	1995–1996–2004–2006, ~ 9.5 years follow-up	288662 men and women, age 50–71 years: 1565 cases	Diabetes, all participants	Yes vs. no	1.41 (1.20–1.66)	Age, sex, race, education, smoking, coffee, BMI, physical activity
				Diabetes duration	None	1.00	
					< 10 years	1.11 (0.89–1.38)	
					≥ 10	1.75 (1.36–2.25)	
				Diabetes, excluding participants with heart disease, stroke, cancer, poor/fair health	Yes vs. no	1.34 (1.06–1.69)	
				Diabetes duration, excluding participants with heart disease, stroke, cancer, poor/fair health	None	1.00	
					≥ 10	1.80 (1.25–2.60)	
					< 10 years	1.00 (0.73–1.38)	
				Diabetes, excluding first five years of follow-up	Yes vs. no	1.33 (1.00-1.78)	
Pupillo E et al, 2016, Italy	700 Italian general practitioners	2013–2013, 1 year follow-up	923356 men and women, age ≥ 18 years: 194 cases	Diabetes	Yes vs.no	1.32 (1.18–1.48)	Age, sex, smoking, obesity, alcohol, hypersomnia, cardiovascular disorders, gastrointestinal disorders, genitourinary infections, restless leg syndrome, antidepressants
Yang YW et al, 2017, Taiwan	Taiwan National Health Insurance Research Database	2000–2006–2011, 7.4 years follow-up	145176 men and women, age ≥ 20 years: 1782 cases	Diabetes mellitus, all	Yes vs. no	1.19 (1.08–1.32)	Age, sex, insurance premium, urbanization level, residential area, type of occupation, comorbidity, flunarizine use, metoclopramide use, zolpidem use, outpatients claim times
				Diabetes mellitus, men	Yes vs. no	1.29 (1.12–1.49)	
				Diabetes mellitus, women	Yes vs. no	1.12 (0.97–1.30)	
				Diabetes mellitus, age 20–39 years	Yes vs. no	0.52 (0.06–4.50)	
				Diabetes mellitus, age 40–64 years	Yes vs. no	1.15 (0.94–1.41)	
				Diabetes mellitus, age ≥ 65 years	Yes vs. no	1.20 (1.06–1.35)	
de Pablo-Fernandez E et al, 2018, England	English national Hospital Episode Statistics	1999–2011, NA	8190323 men and women, age ≥ 25 years: 14252 cases (DM2 only)	Type 2 diabetes	Yes vs. no	1.32 (1.29–1.35)	Age, sex, calendar year of cohort entry, region of residence, Multiple Deprivation score
				Type 2 diabetes, men	Yes vs. no	1.27 (1.23–1.30)	
				Type 2 diabetes, women	Yes vs. no	1.42 (1.37–1.47)	
				Type 2 diabetes, age 25–44 years	Yes vs. no	3.81 (2.84–5.11)	
				Type 2 diabetes, age 45–64 years	Yes vs. no	1.71 (1.61–1.81)	
				Type 2 diabetes, age 65–74 years	Yes vs. no	1.40 (1.35–1.45)	
				Type 2 diabetes, age ≥ 75 years	Yes vs. no	1.18 (1.14–1.21)	
				Type 2 diabetes, follow-up < 1 year	Yes vs. no	1.44 (1.37–1.52)	
				Type 2 diabetes, > 1 year	Yes vs. no	1.29 (1.26–1.33)	
				Type 2 diabetes, 1–4 years	Yes vs. no	1.30 (1.26–1.34)	
				Type 2 diabetes, 5–9 years	Yes vs. no	1.28 (1.23–1.33)	
				Type 2 diabetes, ≥ 10 years	Yes vs. no	1.32 (1.19–1.46)	
				Type 2 diabetes, complications	Yes vs. no	1.49 (1.42–1.56)	
				Type 2 diabetes, no complications	Yes vs. no	1.30 (1.27–1.33)	
Lee SE et al, 2018, South Korea	Korean National Health Insurance database	2005–2008–2013, ~ 7.1 years follow-up	14912368 men and women, age ≥ 30 years: 34834 cases	Diabetes, all	No	1	Age, sex, BMI, smoking, alcohol, exercise, hypertension, dyslipidemia, end-stage renal disease, peripheral artery disease, glucose, insulin use
					DM without DR	1.33 (1.29–1.38)	
					DM with DR	1.75 (1.64–1.86)	
				Diabetes, men	No	1	
					DM without DR	1.27 (1.21–1.34)	
					DM with DR	1.60 (1.45–1.78)	
				Diabetes, women	No	1	
					DM without DR	1.39 (1.33–1.46)	
					DM with DR	1.85 (1.70–2.02)	
				Diabetes, age 30–39 years	No	1	
					DM without DR	1.04 (0.62–1.76)	
					DM with DR	2.09 (0.44–9.99)	
				Diabetes, age 40–59 years	No	1	
					DM without DR	1.11 (1.01–1.24)	
					DM with DR	1.53 (1.23–1.89)	
				Diabetes, age ≥ 60 years	No	1	
					DM without DR	1.35 (1.30–1.40)	
					DM with DR	1.72 (1.60–1.84)	
Kummer BR et al, 2019, USA	Medicare beneficiaries	2008–2015, 5.2 years follow-up	1035536 men and women, mean age 75.9 years: 15531 cases	Diabetes mellitus	Yes vs. no	1.17 (1.11–1.24)	Age, sex, race/ethnicity, Charlson comorbidities
Azizova TV et al, 2019, Russia	Mayak Production Association	1948–1982–2013, 25.5 years follow-up	22377 men and women, mean age 24.1/27.3 years: 300 cases	Diabetes mellitus, men	Yes vs. no	1.82 (1.16–2.74)	Age, sex
				Diabetes mellitus, women	Yes vs. no	1.96 (1.16–3.16)	
Kizza J et al, 2019, China	China Kadoorie Biobank study	2008–2014 - NA, 11.5 years follow-up	480950 men and women, age 30–79 years: 521 cases	Diabetes	Yes vs. no	0.93 (0.67–1.29)	Age, sex, region, income, education, occupation, alcohol, physical activity, smoking, BMI, SBP, DBP
Rhee SY et al, 2020, South Korea	Korean National Health Insurance Database	2009–2010–2016, 3.2 years follow-up	8443351 men and women, age ≥ 40 years: 31577 cases	Diabetes	Yes vs. no	1.37 (1.34–1.41)	Age, sex, BMI, smoking, drinking, physical activity
				Diabetes	No	1	
					IFG	1.04 (1.01–1.07)	
					DM, < 5 years DM, ≥ 5 years	1.19 (1.14–1.23)	
						1.62 (1.57–1.67)	
Jacobs BM et al, 2020, United Kingdom	UK Biobank	2006–2010 - NA, NA	501682 men and women, age 40–69 years: 1276 cases	Diabetes	Yes vs. no	1.27 (1.03–1.57)	Age, sex, deprivation, ethnicity
Sanchez-Gomez A et al, 2021, Spain	The Information System for Research in Primary Care (SIDIAP)	2006–2018, 7.3 years follow-up	3104460 men and women, age 40–80 years: 13715 cases	Type 2 diabetes, all	No	1	Age, sex, BMI, smoking status, socioeconomic status
					Prediabetes	1.07 (1.00-1.14)	
					DM2	1.19 (1.13–1.25)	
				Type 2 diabetes, men	No	1	
					Prediabetes	1.12 (1.03–1.22)	
					DM2	1.27 (1.18–1.38)	
				Type 2 diabetes, women	No	1	
					Prediabetes	1.01 (0.99–1.10)	
					DM2	1.11 (1.04–1.20)	

BMI = Body mass index, DBP = diastolic blood pressure, DR = diabetic retinopathy, IFG = impaired fasting glucose, NA = not available, SBP = systolic blood pressure

**Fig. 2:**
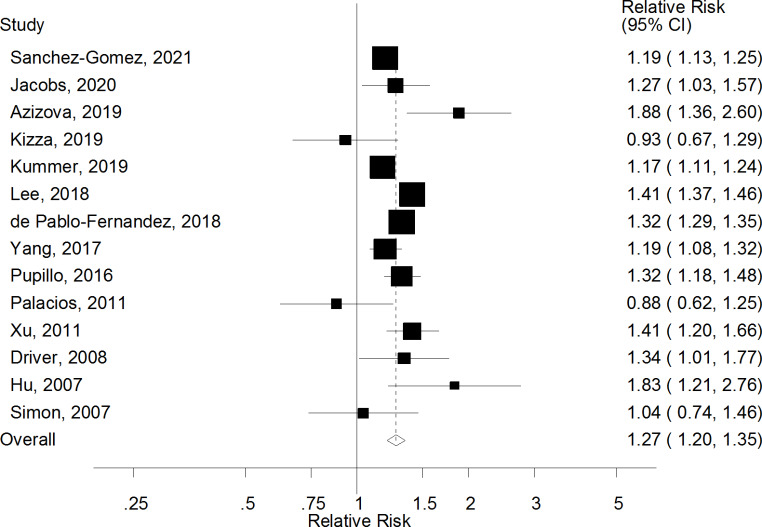
Diabetes mellitus and Parkinson’s disease

The summary RR for PD was 1.27 (95% CI: 1.20–1.35, I^2^ = 82.3%, p_heterogeneity_<0.0001) for persons with diabetes vs. persons without diabetes (Fig. 2). There was no evidence of publication bias with Egger’s test (p = 0.41), Begg’s test (p = 0.99), or by visual inspection of the funnel plot (Supplementary Fig. 1). The summary RR ranged from 1.25 (95% CI: 1.18–1.33) when excluding the study by Lee et al. et al [16] to 1.29 (95% CI: 1.22–1.37) when excluding the study by Kummer et al. [17] (Supplementary Fig. 2). The E-value for the summary RR was 1.86 (lower CI: 1.69), which is the minimum strength that an unmeasured confounder would have with both DM and PD to fully explain away the observed association.

Two studies [21, 26] with 11,547,811 participants and 45,292 cases were included in the analysis of prediabetes and risk of PD. The summary RR was 1.04 (95% CI: 1.02–1.07, I^2^ = 0%, p_heterogeneity_=0.44) (Supplementary Fig. 3). The E-value for the summary RR was 1.24 (lower CI: 1.16).

### Subgroup analyses

Positive associations were consistently observed between DM and PD across most subgroup analyses including analyses stratified by sex, duration of follow-up, exclusion of early follow-up, geographic location, number of cases, type of diabetes, assessment of Parkinson’s disease diagnosis, assessment of diabetes, timing of diabetes diagnosis, diabetes duration, diabetes complications, study quality, and adjustment for confounding factors including age, socioeconomic status, alcohol intake, smoking, and BMI, however, there were few studies in some subgroups (Table 2). There was some indication of a stronger association among diabetes patients with history of complications than for patients without complications (summary RR, 95% CI: 1.54, 1.32–1.80 vs. 1.26, 1.16–1.38), although the test for heterogeneity between subgroups was not significant (p = 0.18). There was relatively high heterogeneity in the overall analysis (I^2^ = 81.8%), but lower or moderate heterogeneity was observed among men, US studies, studies using both prevalent and incident DM cases, and in studies that adjusted for hypertension and serum cholesterol levels (Table 2).

**Table 2:** Subgroup analyses of diabetes mellitus and Parkinson’s disease

		Diabetes mellitus and Parkinson’s disease					
		*n*	Relative risk (95% CI)	*I*^*2*^ (%)	*P*_h_^1^	*P*_h_^2^	References
All studies		14	1.27 (1.20–1.35)	82.3	< 0.0001		8–21
Sex							
Men		8	1.29 (1.24–1.34)	31.1	0.18	0.49/ 0.93^3^	9,10,12,14–16,19,21
Women		7	1.31 (1.17–1.47)	90.3	< 0.0001		9,12,14–16,19,21
Men and women		6	1.23 (1.12–1.35)	52.5	0.06		8,11,13,17,18,20
Follow-up							
<5 years		1	1.32 (1.18–1.48)			0.57	13
5-<10 years		7	1.27 (1.20–1.35)	89.1	< 0.0001		11,14–17,20,21
10-<15 years		2	0.91 (0.71–1.15)	0	0.82		12,18
15-<20 years		2	1.36 (0.78–2.37)	76.7	0.04		8,9
≥20 years		2	1.57 (1.13–2.19)	58.3	0.12		10,19
≥15 years		4	1.47 (1.12–1.92)	60.9	0.05	0.79	8,9,10,19
Excluding early follow-up							
Yes		3	1.33 (1.18–1.50)	23.0	0.27	0.44	9,11,15
No		11	1.24 (1.14–1.35)	85.6	< 0.0001		8,10,12–14,16–21
Geographic location							
Europe		5	1.29 (1.19–1.38)	75.4	0.003	0.98	9,13,15,20,21
America		5	1.20 (1.05–1.36)	53.9	0.07		8,10–12,17
Asia		4	1.31 (1.11–1.55)	84.3	< 0.0001		14,16,18,19
Number of cases							
Cases < 500		2	1.52 (1.08–2.14)	75.5	0.04	0.60	13,19
Cases 500-<1000		5	1.15 (0.90–1.46)	60.8	0.04		8–10,12,18
Cases ≥ 1000		7	1.27 (1.20–1.35)	89.1	< 0.0001		11,14–17,20,21
Parkinson's disease assessment							
No validation or independent assessment		8	1.29 (1.21–1.37)	85.3	< 0.0001	0.71	13–20
Validated or independent assessment		6	1.25 (1.09–1.43)	58.8	0.03		8–12,21
Diabetes type							
Any diabetes		10	1.26 (1.14–1.38)	83.7	< 0.0001	0.72	9,10,15,21
Type 2 diabetes		4	1.29 (1.17–1.41)	81.5	0.001		8,11–14,16–20
Assessment of diabetes							
Self-reported		4	1.34 (1.05–1.72)	69.8	0.02	0.58	10–12,19
Self-reported and validated		1	1.04 (0.74–1.46)				8
Self-report and medical records or fasting glucose		9	1.26 (1.19–1.34)	86.9	< 0.0001		9,13–18,20,21
Timing of diabetes diagnosis							
Prevalent (baseline)		10	1.31 (1.23–1.40)	81.8	< 0.0001	0.21	9,11–13,15,16,18–21
Prevalent and incident		3	1.17 (1.11–1.24)	0	0.51		8,10,17
Incident (newly diagnosed)		1	1.19 (1.08–1.32)				14
Diabetes duration							
<5 years		2	1.38 (0.95-2.00)	83.0	0.02	0.89	10,26
≥5 years		2	1.32 (0.99–1.77)	66.0	0.09		10,26
Diabetes complications							
Yes		3	1.54 (1.32–1.80)	89.6	< 0.0001	0.18	10,15,16
No		3	1.26 (1.16–1.38)	86.3	0.0007		10,15,16
Study quality, modified NOS scale							
0–3 stars		0				0.76	
>3–6 stars		10	1.26 (1.17–1.36)	72.0	< 0.0001		9,14,16,21
>6–8 stars		4	1.30 (1.14–1.48)	92.2	< 0.0001		8,10–13,15,17–20
Adjustment for confounding factors							
Age	Yes	14	1.27 (1.20–1.35)	82.3	< 0.0001	NC	8–21
	No	0					
Education	Yes	4	1.20 (0.89–1.63)	75.6	0.006	0.76	9,11,12,18
	No	10	1.28 (1.20–1.35)	85.3	< 0.0001		8,10,13–17,19–21
Alcohol	Yes	6	1.28 (1.13–1.46)	68.0	0.008	0.64	9,10,12,13,16,18
	No	8	1.25 (1.17–1.34)	80.2	< 0.0001		8,11,14,15,17,19–21
Smoking	Yes	9	1.27 (1.15–1.40)	82.7	< 0.0001	0.99	8–13,16,18,21
	No	5	1.27 (1.16–1.39)	83.0	< 0.0001		14,15,17,19,20
BMI or obesity	Yes	7	1.31 (1.19–1.45)	84.4	< 0.0001	0.42	9–11,13,16,18,21
	No	7	1.23 (1.13–1.35)	79.8	< 0.0001		8,12,14,15,17,19,20
Physical activity	Yes	6	1.29 (1.12–1.50)	65.8	0.01	0.40	9–12,16,18
	No	8	1.25 (1.17–1.33)	79.6	< 0.0001		8,13–15,17,19–21
Hypertension	Yes	2	1.41 (1.37–1.45)	0	0.72	0.20	10,16
	No	12	1.25 (1.17–1.33)	76.4	< 0.0001		8,9,11–15,17–21
Serum cholesterol	Yes	2	1.51 (1.12–2.03)	33.3	0.22	0.33	10,11
	No	12	1.26 (1.19–1.34)	84.5	< 0.0001		8,9,12–21
Coffee or caffeine	Yes	3	1.31 (0.92–1.86)	75.3	0.02	0.70	9,11,12
	No	11	1.27 (1.19–1.34)	84.7	< 0.0001		8,10,13–21

*n* denotes the number of risk estimates (one publication reported a combined risk estimate for two studies), BMI, body mass index, NC, not calculable because no studies were present in one of the subgroups

^1^P for heterogeneity within each subgroup

^2^P for heterogeneity between subgroups with meta-regression analysis

^3^P for heterogeneity between men and women (excluding studies with both sexes) with meta-regression analysis

### Grading of evidence

We considered the overall grading of the evidence to be supportive of a probably causal relationship between DM and PD. This was based on the following criteria which we considered as met: (1) evidence from at least two independent cohort studies (15 cohort studies included); (2) no substantial unexplained heterogeneity relating to the presence or absence of an association, or direction of effect, as the vast majority of studies reported positive associations, no studies reported significantly reduced risk, and there was lower heterogeneity in some subgroup analyses; (3) good quality studies to exclude with confidence the possibility that the observed association results from random or systematic error, including confounding, measurement error and selection bias (i.e. there was little difference in the association in subgroup analyses stratified by risk of bias of the studies, by various adjustments, and the association persisted among studies with more comprehensive assessment of diabetes status vs. only self-reported diabetes status); and (4) evidence for biological plausibility (see section on mechanisms in the discussion).

## Discussion

This systematic review and meta-analysis of fifteen cohort studies with 86,345 PD cases and 29.9 million participants, provides strong epidemiological evidence of a 27% increase in the relative risk of developing PD among DM patients, compared to persons without DM. Persons with prediabetes had a 4% increase in risk, though this latter finding was based on two studies only. The increased risk of PD in persons with DM was observed in men and women, across geographic regions, and strata of other study characteristics (number of cases, type of diabetes, assessment of diabetes, timing of diabetes diagnosis, study quality, and adjustment for most confounding factors).

These findings are in line with previous meta-analyses of five and seven cohort studies, of much smaller sample sizes, which reported a 37–38% increase in risk of PD among diabetes patients [22, 23], and two more recent meta-analyses including 7 and 10 cohort studies which both reported a 29% increase in risk of PD among diabetes patients [24, 25]. Therefore, our findings provide more robust epidemiological evidence on this association, as our analysis was based on roughly twice the number of studies and a much larger sample size and number of PD cases. The findings are also consistent with a recent Mendelian randomization study which found an increased risk of Parkinson’s disease with genetically determined diabetes mellitus [24], providing some support for causality. Our results are not concordant with the findings of case-control studies [23], which are known to be more prone to survival, recall and selection biases, as well as reverse causality. Increased mid-life and early late-life mortality of DM patients may, indeed, underpin the discrepancies between case-control and cohort studies. A “reverse causation” bias may occur in observational studies, related to the long prodromal period of PD, potentially spanning over two decades. This period is known to be associated with progressive accumulation of PD neuropathology, including inclusions of Lewy bodies (LB) and dopaminergic nigrostriatal neuronal loss, known to potentially affect glycaemic control [10], as well as the occurrence of subtle motor and non-motor clinical manifestations that may alter physical activity and diet, both key lifestyle factors in the management of DM. The observation of considerable weight loss in PD patients, seen several years before diagnosis and persisting for several years after diagnosis [36], may also impact diabetes risk favorably, and could potentially explain the observed protective association of DM on PD in case-control studies. Interestingly, in the current analysis based on cohort studies, the positive association between DM and PD persisted in studies with ≥ 15 or ≥ 20 years of follow-up, and was if anything slightly stronger among studies with long durations of follow-up than short follow-up, suggesting that reverse causation is less likely to explain the findings.

The main limitations of studies employing meta-analysis methods involve factors that may potentially affect results, such as potentially unaccounted and residual confounders, sub-optimal study quality, heterogeneity, misclassification or misdiagnosis of diabetes status, as well as publication bias. For instance, pesticide exposure and head trauma are rarely accounted for, in observational cohort studies; additionally diabetes patients have a higher prevalence of overweight/obesity and smoking, and lower levels of physical activity [37], compared to persons without diabetes; therefore, confounding from some of these factors could have impacted the observed results. In our stratified analyses, the associations persisted among studies which adjusted for these and several other factors, making this a less likely explanation for the observed results. In addition, there was no evidence of heterogeneity between the subgroups stratified by various adjustments with meta-regression analyses, however, given the few studies in some of the subgroups it is also possible that we may have been underpowered to detect significant differences between some subgroups. The calculated E-values suggest that such a confounder would have to be relatively strongly associated (RR = 1.86, lower CI: 1.69) with both DM and PD to fully explain away the observed association. However, the E-value for the association between prediabetes and PD was weaker (RR = 1.24, lower CI: 1.16), suggesting the association may have been more vulnerable to confounding.

In terms of study quality, although the mean study quality across studies was graded as moderate, the summary estimates were similar to the subgroup of studies with high study quality. One common contributor to lower than optimal study quality scores was a lack of reporting on adequacy or completeness of follow-up. However, a low score on this point may not necessarily have been a source of bias, but rather due to poor reporting, because many studies have nearly complete follow-up data, facilitated by linkages to health or mortality registries. Other contributors to non-optimal quality scores included features such as non-optimal assessment of exposure and outcome (e.g. using only self-report or registry linkages without independent assessment or validation) and inadequate adjustment for confounders. Nevertheless, the summary estimates persisted in most subgroup analyses stratified by adjustment for various confounding factors.

Although there was high heterogeneity in the overall analysis as measured by the I^2^-value, 12 of 14 risk estimates were in the direction of increased risk and 11 of the risk estimates were statistically significant (95% CIs excluding the null value). Moreover, none of the cohort studies reported a statistically significant reduction in relative risk. Hence, the observed heterogeneity is more likely driven by differences in the strength of the association, than by differences in the direction of the association. Also, several of the included studies were very large and had rather narrow 95% CIs around the risk estimates, but with different sizes of risk estimates, and thus, the 95% CIs for the different studies did not always overlap. This likely explains the high I^2^-value in spite of the relative consistency of the direction of the association, thus less problematic than the presence of heterogeneity related to the direction of an association. In our subgroup analyses, there was less heterogeneity in studies of men or men and women combined, and among US studies.

Diabetes was self-reported in some studies and this may have led to misclassification of diabetes status that could, potentially, result in an underestimation of the association between DM and PD. However, we found little difference in the summary estimates between subgroups of studies that only relied on self-report and those that used a combination of self-report and linkages to medical records or had a measure of fasting blood glucose at baseline. Subgroup analyses stratified by whether studies only used prevalent (baseline) diabetes cases or also included incident diabetes cases, did not show significant heterogeneity between subgroups, although the summary estimate was slightly higher for the former than the latter (1.30 vs. 1.17). Misclassification of diabetes diagnoses by using only prevalent cases could result in regression dilution bias (bias to the null) as the lack of information on incident diabetes cases could lead to misclassification and potential underestimation of the association. However, this would depend on a recent diabetes diagnosis being most relevant to Parkinson’s disease development. Given that we observed the strongest association in the subgroup using prevalent diabetes cases only, it is possible that long-standing diabetes may be of greater importance. Survival bias is less likely to explain our findings because if such bias was present one would expect a weaker association in the studies using only prevalent diabetes cases, than in those also using incident cases. Although we found no significant difference in the results when stratified by the reported diabetes duration, this analysis was based on only two studies. The summary estimates were somewhat stronger for participants with diabetes complications than among those without such complications (summary RR, 95% CI = 1.54, 1.32–1.80 vs. 1.26, 1.16–1.38). As diabetes complications typically occur in patients with longer disease duration or poor glycaemic control, it is plausible that a longer disease duration and poor diabetes management are key driving factors for the stronger association with PD risk. Given the limited number of studies in these subgroup analyses and the lack of consistent cut-offs reported for diabetes duration, further epidemiological and mechanistic studies are warranted to elucidate the precise role of disease duration and glycaemic control on PD risk. In addition, the available studies did not investigate the role of specific diabetes medications on the observed risk. The majority of the cohort studies included in our meta-analysis only reported on DM overall, three studies reported on type 2 diabetes (T2D) only and none on type 1 diabetes. Given that the vast majority (over 90%) of diabetes cases are of type 2 (1), the current findings likely reflect the impact of insulin resistance and T2D. Lastly, we explored potential publication bias, but found no evidence of such bias with the statistical tests or by inspection of the funnel plot.

Several biological mechanisms may contribute to the increased risk of PD in patients with diabetes. Hyperglycemia, resulting from hypoinsulinaemia in type 1 diabetes or insulin resistance (IR) in T2D, exposes neurons to increased metabolic stress, neuronal dysfunction and death, thus directly contributing to PD pathogenesis [38]. Experiments in diabetic mice showed reduced dopamine transporters [39] and dopamine levels in the striatum [40], thus increasing the vulnerability of nigro-striatal neurons. Recent experiments, using diabetes-induced MitoPark mice, showed that the acquisition of IR phenotype in these animals results in mitochondrial dysfunction by suppressing PGC-1α expression, promoting the upregulated ROS production and oxidative stress, as well as the upregulated expression of phosphorylated α-synuclein (SNCA) [41], a key constituent of Lewy bodies (LB) [42]. Mitochondrial dysfunction, leading to neuronal death was also a main finding of studies using knockout of insulin receptor (NIRKO) mice [43] and diabetic db/db mice [44]. Increased accumulation and phosphorylation of α-synuclein was also observed within the cortex, pre-commissural putamen and dopaminergic neurons in the substantia nigra of cynomolgus monkeys, with spontaneous T2D-like pathology [45]. Of note, abnormal SNCA and LB burden are key pathological features in PD.

A direct effect of hyperglycemia is the increase of advanced glycation end-products (AGEs) and glycation agents, such as the highly reactive methylglyoxal (MGO), that may reach particularly high levels in the substantia nigra [46]. The interactions of AGEs with their receptors (RAGE) may lead to oxidative stress, inflammation and cell death [47]. AGEs have been found in LBs and have been shown, in vitro studies, to cross-link with SNCA to induce its aggregation and formation of SNCA oligomers, of higher neurotoxicity [48]. Furthermore, MGO may inhibit SNCA degradation and increase its accumulation and also react with dopamine to form 1-acetyl-6,7 dihysroxy-1.2.3.4-tetrahysroisoquinoline (ADTIQ), that may further contribute to dopaminergic degeneration [48].

In view of the potential increase of PD risk in diabetes patients, repurposing of antidiabetic medications for the treatment of PD is gaining increasing research interest, in the absence of effective disease modifying therapies. Recently, Mor et al. have demonstrated the neurotoxic effect of bcat-1 knockdown, in an animal model known to recapitulate PD features [49]. Neurotoxicity was found to be mediated through increased mitochondrial respiration (or “hyperactivity”) and oxidative damage. The authors showed that administration of metformin, a first- line anti-T2D medication, reduced mitochondrial respiration to control levels and significantly improved both motor function and neuronal viability [49]. Metformin was also shown to improve motor functions in 6-hydroxydopamine (6-OHDA)-lesioned mice, by activating the AMPK and BDNF signaling pathways and regulating or suppressing genes in reactive astrocytes [50]. In human trials, a double blind placebo-controlled study of Exenatide, a GLP-1 receptor agonist, showed a beneficial effect in PD patients [51]. In a subsequent study, evaluating target engagement through neuronal derived exosomal vesicles (NEVs) isolated from serum samples, it has been shown that patients with exenatide had an increased protein activation of Akt and mTOR cascades, compared to placebo [50].

Strengths of this meta-analysis include the population-based cohort design of the included studies, mitigating the potential for survival bias, recall bias, selection bias, and reverse causation, which may affect case-control studies to a larger degree; secondly it’s large sample size of 29.9 million participants, including over 86,000 PD cases, providing statistical power to detect a moderate association; finally the robustness of the findings in multiple subgroup and sensitivity analyses.

As the ever increasing numbers of persons with diabetes are projected to reach 700 million by 2025 worldwide [1], increased rates of debilitating late-onset neurodegenerative diseases such as AD, PD, as well as the associated forms of dementia with Lewy bodies, and Parkinson’s disease dementia are set to become another consequence of the diabetes epidemic, adding significant healthcare and socio-economic burden worldwide. Although the observed association between DM and PD is of moderate size, the findings are still likely to have important public health implications because of the large number of persons who live with diabetes worldwide. In this context, our findings strongly support the need for urgent global public health measures to effectively address the diabetes epidemic worldwide, that may have the added significant benefit in preventing PD, AD and related late-onset neurodegenerative diseases.

## Conclusion

This systematic review and meta-analysis provides epidemiological evidence that patients with diabetes mellitus have a 27% increase in the relative risk of PD compared to persons without diabetes. The likelihood of causality was graded as probable using WCRF criteria. There was a suggestion of a 4% increase in risk of PD among those with prediabetes. Further studies should aim at elucidating the specific contribution of disease duration and age of diabetes onset, longitudinal glycaemia and its variability, as well as diabetic complications and antidiabetic medications in this association.

## Electronic supplementary material

Below is the link to the electronic supplementary material.

Supplementary Material 1

Supplementary Material 2
