# Supplementary material for: Diabetes mellitus, prediabetes and the risk of Parkinson’s disease: a systematic review and meta-analysis of 15 cohort studies with 29.9 million participants and 86,345 cases
Source: Eur J Epidemiol. 2023 Apr 25;38(6):591–604. doi: 10.1007/s10654-023-00970-0 (PMC10232631; doi:10.1007/s10654-023-00970-0)
Supplement: Supplementary file 2 — Supplementary Material 2 [file 10654_2023_970_MOESM2_ESM.pdf]

Supplement for:

Aune D, Schlesinger S, Mahamat-Saleh Y, Zheng B, Udeh-Momoh CT, Middleton LT. Diabetes mellitus, pre-diabetes and the risk of Parkinson’s disease: a systematic review and meta-analysis of 15 cohort studies with 29.9 million participants and 86,345 cases. European Journal of Epidemiology
https://doi.org/10.1007/s10654-023-00970-0

Supplementary Figure 1. Funnel plot of diabetes mellitus and Parkinson's disease

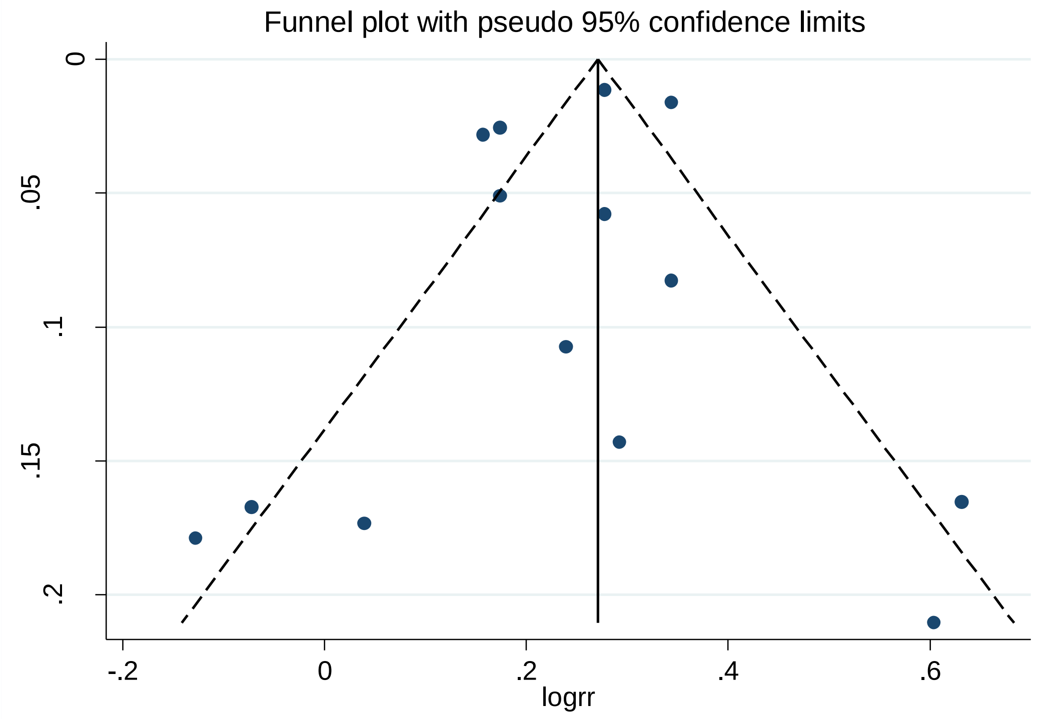

Supplementary Figure 2. Influence analysis of diabetes mellitus and risk of Parkinson's disease

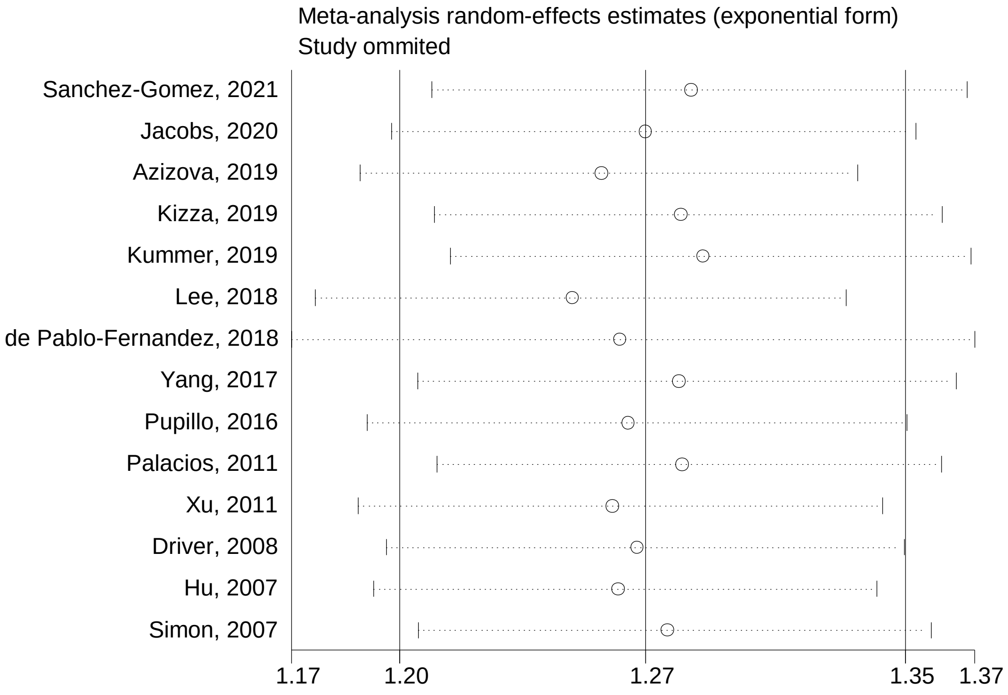

Study omitted	e^coef.	[95% Conf.	Interval]
Sanchez-Gomez, 2021	1.286213	1.2110984	1.3659863
Jacobs, 2020	1.2729714	1.1993399	1.3511233
Azizova, 2019	1.2602396	1.1903292	1.3342559
Kizza, 2019	1.2831489	1.211754	1.3587502
Kummer, 2019	1.289562	1.2163094	1.3672262
Lee, 2018	1.2517939	1.1773205	1.330978
de Pablo-Fernandez, 2018	1.2654898	1.1704093	1.3682944
Yang, 2017	1.2825159	1.2068717	1.3629012
Pupillo, 2016	1.2680068	1.1922166	1.3486149
Palacios, 2011	1.2835622	1.2125785	1.3587011
Xu, 2011	1.2633793	1.1897087	1.3416119
Driver, 2008	1.2705973	1.197821	1.3477954
Hu, 2007	1.2649577	1.1941773	1.3399332
Simon, 2007	1.2791802	1.2070223	1.3556517
Combined	1.2728658	1.2016861	1.3482618

Supplementary Figure 3. Prediabetes and Parkinson's disease

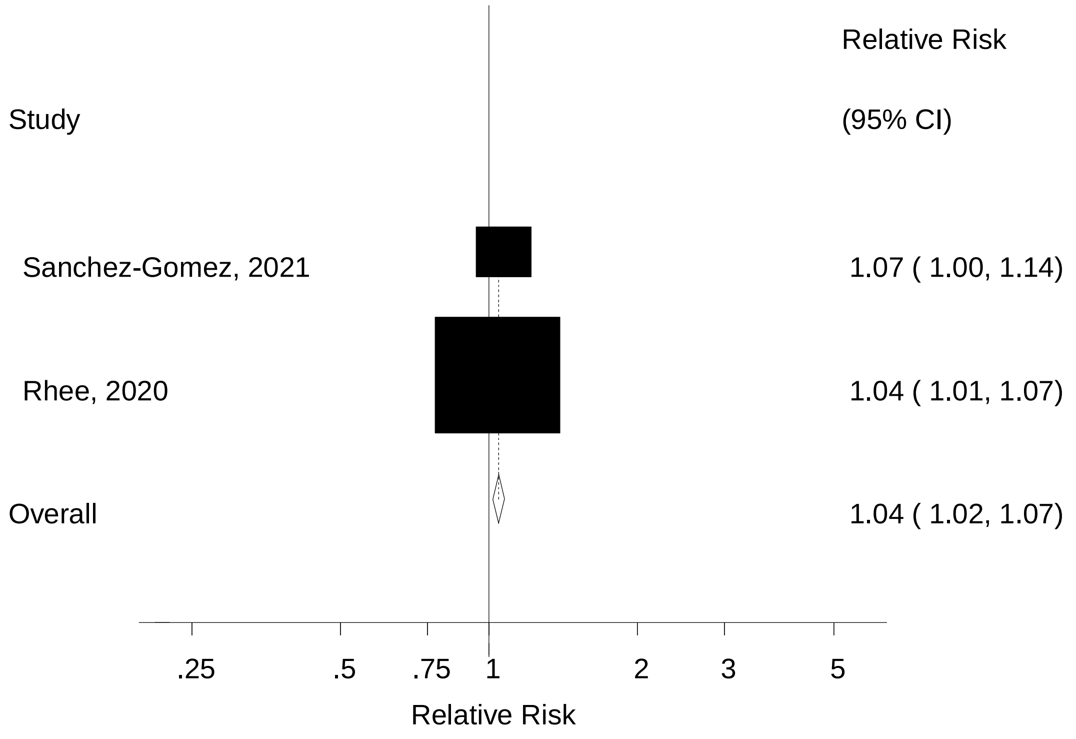

Supplementary text

PubMed search terms

(diabetes OR diabetes[MeSH] OR "blood glucose" OR blood glucose[MeSH] OR "plasma glucose" OR plasma glucose[MeSH] OR "serum glucose" OR serum glucose[MeSH] OR "medical history" OR medical history[MeSH])

AND

("Parkinson’s" OR Parkinson’s[MeSH] OR "Parkinson")

AND

("case-control" OR cohort OR prospective OR longitudinal OR retrospective OR "follow-up" OR "cross-sectional" OR "hazard ratio" OR "hazard ratios" OR "relative risk" OR "relative risks" OR "incidence rate ratio" OR "incidence rate ratios" OR "odds ratio" OR odds ratios OR incidence)

Embase search terms

((diabetes or blood glucose or serum glucose or plasma glucose or medical history).ab,ti.

OR diabetes/ or blood glucose/ or serum glucose/ or plasma glucose/ or medical history/)

AND

((Parkinson's or Parkinson).ab,ti. OR (Parkinson's/ or Parkinson/))

AND

(case-control or cohort or prospective or longitudinal or retrospective or follow-up or cross-sectional or hazard ratio or hazard ratios or relative risk or relative risks or incidence rate ratio or incidence rate ratios or odds ratio or odds ratios or incidence).af.

Supplementary Table 1. List of excluded studies and exclusion reasons

Exclusion reason	Reference number
Abstract	(1-30)
Case-control study	(31-55)
Case only study	(56-59)
Comment, editorial, letter	(60-74)
Cross-sectional study	(75-84)
Duplicate	(85-91)
Genetic study	(92)
Meta-analysis	(93-101)
No risk estimates	(102-104)
Not relevant data	(105-114)
Not relevant exposure	(115-167)
Not relevant outcome	(168-173)
Not retrieved	(174)
Patient population	(175-177)
Progression of Parkinson's disease	(178-184)
Review	(185-201)

Reference List

(1) Palacios N, McCullough M, Jacobs E, Patel A, Mayo T, Schwarzschild M, Ascherio A. Obesity, diabetes and risk of Parkinson Disease in a large prospective Cohort of men and women. Neuroepidemiology Conference: Regional North American Annual Meeting of the World Federation of Neurology -Research Group on Neuroepidemiology Seattle, WA United States Conference Publication 2009;33(1):70-1.

(2) Xu Q, Park Y, Huang X, Umbach D, Hollenbeck A, Blair A, Schatzkin A, Chen H. Diabetes mellitus and risk of Parkinson's disease. Movement Disorders Conference: 2nd World Parkinson Congress, WPC Glasgow United Kingdom Conference Publication 2010;25(SUPPL. 3):S615.

(3) Papapetropoulos S, Tibbetts A, Barnard J, Lanes AS, Ward A, Mitchels SL, O'Neill GN. Comorbid conditions in patients with Parkinson's disease: A US claims database analysis. Movement Disorders Conference: 14th International Congress of Parkinson's Disease and Movement Disorders Buenos Aires Argentina Conference Publication 2010;25(SUPPL. 2):S261.

(4) Knekt P, Saaksjarvi K, Mannisto S, Rissanen H, Laaksonen MA. Serum triglycerides, related to an elevated type 2 diabetes risk, predict a reduced risk of Parkinson's disease. Journal of Diabetes Conference: 4th International Congress on Prediabetes and the Metabolic Syndrome Madrid Spain Conference Publication 2011;3(SUPPL. 1):122.

(5) Chen H. The shanghai parkinson study: Prospective parkinson research in China. Neuroepidemiology Conference: 2nd International Congress on Neurology and Epidemiology Nice France Conference Publication 2012;39(3-4):214-5.

(6) Doiron M, Simard M. Vascular risk factors and cognition in idiopathic parkinson's disease. Journal of Neuropsychiatry and Clinical Neurosciences Conference: 23rd Annual Meeting of the American Neuropsychiatric Association, ANPA 2012;Spring.

(7) Noyce AJ, Bestwick J, Silveira-Moriyama L, Hawkes CH, Giovannoni G, Lees AJ, Schrag A. Risk factors and early non-motor features for Parkinson's disease: A systematic review and meta-analysis. Movement Disorders Conference: 16th International Congress of Parkinson's Disease and Movement Disorders Dublin Ireland Conference Publication 2012;27(SUPPL. 1):S4.

(8) Ton TG, Jie L, Biggs ML, Delaney JA, Landsittel D, Hu S, Curtis L, Searles NS, Longstreth WT, Checkoway H, Jain S. Cognitive function and risk of parkinson's disease in the cardiovascular health study. Neuroepidemiology Conference: Regional North American Annual Meeting of the World Federation of Neurology - Research Group on Neuroepidemiology 2013;May.

(9) Saaksjarvi K, Knekt P, Mannisto S, Lyytinen J, Laaksonen MA, Heliovaara M. Prospective study on the metabolic syndrome and incidence of parkinson's disease. Journal of Diabetes Conference: 5th International Congress on Prediabetes and the Metabolic Syndrome Early Interventions for Diabetes and Dysglycaemia Surgery in the Treatment of Obesity and Diabetes Vienna Austria Conference Publication 2013;April.

(10) Garcia-Gracia C, Khan T, Reyes D, McMahan S, Galvez-Jimenez N. The prevalence of stroke in parkinson's disease is high: A risk factor assessment. Neurology Conference: 65th American Academy of Neurology Annual Meeting San Diego, CA United States Conference Publication 2013;80(1):12.

(11) Kodama M, Kato S. Diabetes in Parkinson disease, Dementia with Lewy body and Alzheimer disease. Clinical Neurology Conference: 55th Annual Meeting of the Japanese Society of Neurology Japan 2014;54(Supplement 1):S222.

(12) de Pablo-Fernandez E, Sierra-Hidalgo F, Sanchez-Ferro A, ito-Leon J, Bermejo-Pareja F. Diabetes and risk of Parkinson's disease: Evidence from the population-based NEDICES

study. Movement Disorders Conference: 18th International Congress of Parkinson's Disease and Movement Disorders Stockholm Sweden Conference Publication 2014;29(SUPPL. 1):S542.

(13) Dexter DT, Gveric D, Gentleman S, Middleton L, Roncaroli F, Pearce R, Reynolds R. Parkinson's UK Tissue Bank: A unique tissue resource for fostering Parkinson's disease research. Movement Disorders Conference: 18th International Congress of Parkinson's Disease and Movement Disorders Stockholm Sweden Conference Publication 2014;29(SUPPL. 1):S8-S9.

(14) Swallow DMA, Lawton MA, Grosset KA, Malek N, Bajaj N, Barker RA, Ben-Shlomo Y, Burn DJ, Foltynie T, Morris HR, Williams N, Wood NW, et al. Cardiovascular risk and statin use in recent onset Parkinson's disease. Journal of the Neurological Sciences Conference: 22nd World Congress of Neurology, WCN 2015;15.

(15) Lee C-J, Yeh Y-C, Liang C-Y, Chen Y-Y. Risk of Parkinson's disease in patients with diabetes mellitus and other comorbidities in Taiwan. Neurology Conference: 68th American Academy of Neurology Annual Meeting, AAN 2016;05.

(16) Koh E, Lee S, Jang J, Woo C, Lee K-U. Association between diabetic retinopathy and Parkinson's disease: The Korean national health insurance service database. Diabetologia Conference: 54th Annual Meeting of the European Association for the Study Diabetes, EASD 2018;(Supplement 1):October.

(17) Houghton R, Boess F, Verselis L, Ding Y, Freitas R, Constantinovici N, Ong R. Comorbidities and treatment initiation in patients with Parkinson's disease: A retrospective cohort study using a US insurance claims database. Pharmacoepidemiology and Drug Safety Conference: 34th International Conference on Pharmacoepidemiology and Therapeutic Risk Management Czechia 2018;27(Supplement 2):318.

(18) Kim WC. Effect of diabetes mellitus on development of Parkinson's disease. Parkinsonism and Related Disorders Conference: 22nd World Congress of the International Association of Parkinsonism and Related Disorders Vietnam 2018;46(Supplement 2):e78.

(19) Hong CT, Chan L, Bai CH. Prevalence of cerebral and cardiovascular disease in Parkinson's disease patients: A meta-analysis. Parkinsonism and Related Disorders Conference: 22nd World Congress of the International Association of Parkinsonism and Related Disorders Ho Chi Minh City Vietnam 2018;46 (Supplement 2):e9.

(20) Athauda D. Insulin resistance, diabetes and Parkinson's disease - How do they link together? Journal of Parkinson's Disease Conference: 5th World Parkinson Congress, WPC 2019;(1):2019.

(21) Minar M, Dragasek J, Valkovic P. Comorbidities in Parkinson's disease - The results from national epidemiological study cosmos. Journal of the Neurological Sciences Conference: World Congress of Neurology (WCN 2019;(Supplement):15.

(22) Avila RA, Jesus MS, Aguilar M, Planellas L, Caldentey JG, Pons NC, Legarda I, Vara JH, Cubo E, Manzanares LL, Aramburu IG, Alonso MJC, et al. Comorbidity and polypharmacy in Parkinson's disease: Comparative study with control group. results from the COPPADIS study cohort. Neurology Conference: 71st Annual Meeting of the American Academy of Neurology, AAN 2019;(15 Supplement 1):April.

(23) Mekala S, Chandirasekar R, Uthayakumar V, Mathivanan R, Ragadevi M, Anitha E, Loganathan K. Study on the clinical and genetic association in Parkinson's disease risk of diabetes mellitus patients with high cholesterol level. Movement Disorders Clinical Practice Conference: 6th Asian and Oceanian Parkinson's Disease and Movement Disorders Congress China 2019;6(Supplement 1):S70.

(24) Belete D, Jacobs B, Schrag A, Noyce A. Exploring the Parkinson's disease phenome in the UK biobank population. Neurology Conference: 72nd Annual Meeting of the American Academy of Neurology, AAN 2020;(15 Supplement).

(25) Esquivel-Zapata O, Escobar-Valdivia E, Romero JDG, Salas AA, Cervantes A, Violante MR, Sarabia-Tapia C, Lopez-Alamillo S, Rios Y, Cardenas O, bundes-Corona R, Herrera F. Timing of diabetes diagnosis and its influence on the age of Parkinson's disease onset. Movement Disorders Clinical Practice Conference: 3rd Pan American Parkinson's Disease and Movement Disorders Congress United States 2020;7(Supplement 1):S77-S78.

(26) Parra-Lopez G, Bazan-Rodriguez L, Rodriguez-Violante M, Cervantes-Arriaga A, Jorge-de SA, Cruz-Fino D, Pascacio-Astudillo J, Llorens-Arenas R. Impact of cardiovascular risk factors in the evolution and treatment of Parkinson's disease in Mexican population. Movement Disorders Conference: 2020;(Supplement 2):June.

(27) Lee SE, Choi HS, Yoo J, Han K, Kim K-A. The risk of Parkinson's disease according to the diabetic kidney disease status in the Korean population. Journal of Diabetes Investigation Conference: 11th International Congress of Diabetes and Metabolism and the 13th AASD Scientific Meeting Virtual 2021;12 (SUPPL 1):13.

(28) Larsen MEC, Thykjaer AS, Pedersen FN, Rubin KH, Blaabjerg M, Stokholm L, Grauslund J. Diabetic Retinopathy as a Potential Marker of Parkinsons Disease: Results from a Nationwide Register-Based Cohort Study. European Journal of Ophthalmology Conference: 31st Meeting of the European Association for the Study of Diabetes Eye Complications Study Group, EASDec Odense Denmark 2021;30 (2 SUPPL):27-8.

(29) Larsen MEC, Thykjr AS, Pedersen F, Blaabjerg M, Rubin K, Stokholm L, Grauslund J. Diabetic retinopathy as a potential marker of prevalent and incident Parkinson's disease: Results from a nationwide register-based cohort study. Investigative Ophthalmology and Visual Science Conference: Annual Meeting Association for Research in Vision and Ophthalmology, ARVO 2021;(8):June.

(30) Grauslund J, Pedersen HE, Sandvik CH, Subhi Y, Pedersen FN. Relationship between Diabetic Retinopathy and Systemic Neurodegenerative Diseases: A Systematic Review and Meta-Analysis. European Journal of Ophthalmology Conference: 31st Meeting of the European Association for the Study of Diabetes Eye Complications Study Group, EASDec Odense Denmark 2021;30 (2 SUPPL):4-5.

(31) Kessler II. Epidemiologic studies of Parkinson's disease. 3. A community-based survey. Am J Epidemiol 1972 Oct;96(4):242-54.

(32) Wechsler LS, Checkoway H, Franklin GM, Costa LG. A pilot study of occupational and environmental risk factors for Parkinson's disease. Neurotoxicology 1991;12(3):387-92.

(33) Hubble JP, Cao T, Hassanein RE, Neuberger JS, Koller WC. Risk factors for Parkinson's disease. Neurology 1993 Sep;43(9):1693-7.

(34) Morano A, Jimenez-Jimenez FJ, Molina JA, Antolin MA. Risk-factors for Parkinson's disease: case-control study in the province of Caceres, Spain. Acta Neurol Scand 1994 Mar;89(3):164-70.

(35) Maher NE, Golbe LI, Lazzarini AM, Mark MH, Currie LJ, Wooten GF, Saint-Hilaire M, Wilk JB, Volcjak J, Maher JE, Feldman RG, Guttman M, et al. Epidemiologic study of 203 sibling pairs with Parkinson's disease: the GenePD study. Neurology 2002 Jan 8;58(1):79-84.

(36) Powers KM, Smith-Weller T, Franklin GM, Longstreth WT, Jr., Swanson PD, Checkoway H. Diabetes, smoking, and other medical conditions in relation to Parkinson's disease risk. Parkinsonism Relat Disord 2006 Apr;12(3):185-9.

(37) Scigliano G, Musicco M, Soliveri P, Piccolo I, Ronchetti G, Girotti F. Reduced risk factors for vascular disorders in Parkinson disease patients: A case-control study. Stroke 2006;37(5):1184-8.

(38) Ma HI, Kim JH, Chu MK, Oh MS, Yu KH, Kim J, Hahm W, Kim YJ, Lee BC. Diabetes mellitus and drug-induced Parkinsonism: a case-control study. J Neurol Sci 2009 Sep 15;284(1-2):140-3.

(39) D'Amelio M, Ragonese P, Callari G, Di BN, Palmeri B, Terruso V, Salemi G, Famoso G, Aridon P, Savettieri G. Diabetes preceding Parkinson's disease onset. A case-control study. Parkinsonism Relat Disord 2009 Nov;15(9):660-4.

(40) Miyake Y, Tanaka K, Fukushima W, Sasaki S, Kiyohara C, Tsuboi Y, Yamada T, Oeda T, Miki T, Kawamura N, Sakae N, Fukuyama H, et al. Case-control study of risk of Parkinson's disease in relation to hypertension, hypercholesterolemia, and diabetes in Japan. J Neurol Sci 2010 Jun 15;293(1-2):82-6.

(41) Schernhammer E, Hansen J, Rugbjerg K, Wermuth L, Ritz B. Diabetes and the risk of developing Parkinson's disease in Denmark. Diabetes Care 2011 May;34(5):1102-8.

(42) Lai S-W, Su L-T, Lin C-H, Tsai C-H, Sung F-C, Hsieh DPH. Polypharmacy increases the risk of Parkinson's disease in older people in Taiwan: A population-based study. Psychogeriatrics 2011;11(3):150-6.

(43) Savica R, Grossardt BR, Ahlskog JE, Rocca WA. Metabolic markers or conditions preceding Parkinson's disease: a case-control study. Mov Disord 2012 Jul;27(8):974-9.

(44) Cereda E, Barichella M, Cassani E, Caccialanza R, Pezzoli G. Clinical features of Parkinson disease when onset of diabetes came first: A case-control study. Neurology 2012 May 8;78(19):1507-11.

(45) Skeie GO, Muller B, Haugarvoll K, Larsen JP, Tysnes OB. Parkinson disease: associated disorders in the Norwegian population based incident ParkWest study. Parkinsonism Relat Disord 2013 Jan;19(1):53-5.

(46) McLean G, Hindle JV, Guthrie B, Mercer SW. Co-morbidity and polypharmacy in Parkinson's disease: Insights from a large Scottish primary care database. BMC Neurology 2017;17(1):126.

(47) Marko-Kucsera M, Vecsei L, Paulik E. Association of cardiovascular risk factors and Parkinson's disease - case-control study in South East Hungary. Ideggyogy Sz 2018 Jan 30;71(1-02):57-62.

(48) Chen SF, Yang YC, Hsu CY, Shen YC. Risk of Parkinson's disease in patients with hypothyroidism: A nationwide population-based cohort study. Parkinsonism Relat Disord 2020 May;74:28-32.

(49) Cervantes-Arriaga A, Esquivel-Zapata O, Escobar-Valdivia E, Garcia-Romero D, cocer-Salas A, Rodriguez-Violante M. Association between cardiometabolic comorbidities and parkinson's disease in a mexican population. [Spanish]. Gaceta Medica de Mexico 157(6) (pp 645-650), 2021;157(6):645-50.

(50) Venkatesan D, Iyer M, S R.W., G L., Vellingiri B. The association between multiple risk factors, clinical correlations and molecular insights in Parkinson's disease patients from Tamil Nadu population, India. Neuroscience Letters 2021;755:135903.

(51) Cereda E, Barichella M, Cassani E, Caccialanza R, Pezzoli G. Clinical features of Parkinson's disease when onset of diabetes came first: A case control study. Clinical Nutrition, Supplement Conference: 34th European Society for Clinical Nutrition and Metabolism, ESPEN Congress Barcelona Spain Conference Publication 2012;7(1):172.

(52) Rabey J, Abbruzzese G, Prokhorov T, Bonuccelli U. Reduced cardiovascular risk factors and cardiovascular events in idiopathic Parkinson's disease patients compared with controls. Journal of Neurology Conference: 23rd Meeting of the European Neurological Society Barcelona Spain Conference Publication 2013;260(SUPPL. 1):S62.

(53) Pinzon R. The life style and risk of parkinson disease. Movement Disorders Conference: 21st International Congress of Parkinson's Disease and Movement Disorders Canada 2017;32(Supplement 2):12-3.

(54) az-Torne C, Pou MA, Orfila F, Jeria S, Fernandez S, Park H, Castellvi I, Corominas H. Does gout protect from parkinson's disease: A case-control study from an urban population. Annals of the Rheumatic Diseases Conference: Annual European Congress of Rheumatology, EULAR 2018;(Supplement 2):June.

(55) Parra-Lopez G, Bazan-Rodriguez L, Rodriguez-Violante M, Cervantes-Arriaga A, Jorge de SA, Escobar-Barrios M, Carreon-Bautista E. Cardiometabolic syndrome and risk of Parkinson's disease: A casecontrol study in Mexican population. Movement Disorders Conference: 2020;(Supplement 2):June.

(56) Barbeau A, Pourcher E. New data on the genetics of Parkinson's disease. Canadian Journal of Neurological Sciences 1982;9(1):53-60.

(57) Kotagal V, Albin RL, Muller MLTM, Bohnen NI. Cardiovascular Risk Factor Burden in Veterans and Non-Veterans with Parkinson Disease. J Parkinsons Dis 2018;8(1):153-60.

(58) Chung SJ, Jeon S, Yoo HS, Kim G, Oh JS, Kim JS, Evans AC, Sohn YH, Lee PH. Detrimental effect of type 2 diabetes mellitus in a large case series of Parkinson's disease. Parkinsonism Relat Disord 2019 Jul;64:54-9.

(59) Tan EE, Pagano G, Tagliati M. The impact of diabetes mellitus on non-motor symptoms in patients with Parkinson's disease. Movement Disorders Conference: 2019;June.

(60) Ikeda K, Kashihara H, Tamura M, Kano O, Iwamoto K, Iwasaki Y. Body mass index and the risk of Parkinson disease [1]. Neurology 2007;68(24):2156.

(61) Palacios N, Ascherio A. Reply to: Diabetes and risk of Parkinson's disease. Movement Disorders 2013;28(2):258.

(62) Kawada T. Risk reduction of Parkinson disease by statin therapy in patients with diabetes. Annals of Neurology 2017;81(1):157.

(63) Rhee SY, Lee WY. Association Between Glycemic Status and the Risk of Parkinson Disease: A Nationwide Population-Based Study. Diabetes Care 2020;43:2169-2175. Diabetes Care 2021 May;44(5):e97.

(64) Melgoza IP, Jilani R, Shehzad Z, Anam AK. Comment on Rhee et al. Association Between Glycemic Status and the Risk of Parkinson Disease: A Nationwide Population-Based Study. Diabetes Care 2020;43:2169-2175. Diabetes Care 2021 May;44(5):e95-e96.

(65) Ganesh A, Galetta S. Editors' note: Association between diabetes and subsequent Parkinson disease: A record-linkage cohort study. Neurology 2019;92(19):924.

(66) Foltynie T, Athauda D. Diabetes, BMI, and Parkinson's. Movement Disorders 2020;35(2):201-3.

(67) Wilson C. Epidemiology: Parkinson disease and diabetes. Nat Rev Endocrinol 2011 Jun;7(6):316.

(68) Kotagal V, Albin RL, Muller ML, Bohnen NI. Clinical features of Parkinson disease when onset of diabetes came first: a case-control study. Neurology 2012 Oct 23;79(17):1835-6.

(69) Kawabe K, Ikeda K, Iwasaki Y. Clinical features of Parkinson disease when onset of diabetes came first: a case-control study. Neurology 2012 Oct 23;79(17):1835.

(70) Cereda E, Barichella M, Pedrolli C, Klersy C, Cassani E, Caccialanza R, Pezzoli G. Diabetes and risk of Parkinson's disease. Mov Disord 2013 Feb;28(2):257.

(71) de Pablo-Fernandez E, Warner T. Author response: Association between diabetes and subsequent Parkinson disease: A record-linkage cohort study. Neurology 2019 May 7;92(19):925-6.

(72) Lai SW. Reader response: Association between diabetes and subsequent Parkinson disease: A record-linkage cohort study. Neurology 2019 May 7;92(19):925.

(73) Melgoza IP, Jilani R, Shehzad Z, Anam AK. Association between glycemic status and the risk of parkinson disease: A nationwide population-based study. diabetes care. Diabetes Care 2021;44(5):e95-e96.

(74) Giuntini M, Baldacci F, Del PE, Bonuccelli U, Ceravolo R. Diabetes is associated with postural and cognitive domains in Parkinson's disease. Results from a single-center study. Parkinsonism and Related Disorders 2020;(6):June.

(75) Schelp AO, Mendes-Chiloff CL, Bazan R, Paduan VC, Pioltini ABM. Metabolic syndrome and dementia associated with Parkinson's disease: Impact of age and hypertension. Arquivos de Neuro-Psiquiatria 2012;70(2):114-8.

(76) Liu H, Xie Y-M, Yi D-H, Wang Y-Y. Clinical and medicine characteristics of patients with Parkinson s syndrome. [Chinese]. Zhongguo Zhongyao Zazhi 2014;39(18):3493-8.

(77) Klimek P, Kautzky-Willer A, Chmiel A, Schiller-Fruhwirth I, Thurner S. Quantification of diabetes comorbidity risks across life using nation-wide big claims data. PLoS Comput Biol 2015 Apr;11(4):e1004125.

(78) de Pablo-Fernandez E, Sierra-Hidalgo F, ito-Leon J, Bermejo-Pareja F. Association between Parkinson's disease and diabetes: Data from NEDICES study. Acta Neurol Scand 2017 Dec;136(6):732-6.

(79) Salari M, Mirmosayyeb O, Etemadifar M, Shaygannejad V, Khorvash F, Najafi MR, Ashtari F, Chitsaz A. Demographic features and clinical characteristics of patients with Parkinson's disease in Isfahan, Iran. Iranian Journal of Neurology 2018;17(1):6-10.

(80) Barrios ME, Gonzalez-Hernanez V, Perez-Lohman C, rredondo-Blanco K, Garcia NO, Vargas-Jaramillo JD, Zeron-Martinez R, Cervantes-Arriaga A, Rodriguez-Violante M. Chronic degenerative diseases and the risk of Parkinson's disease: A case-control study in Mexican population. Movement Disorders Conference: 2nd Pan American Parkinson's Disease and Movement Disorders Congress Miami, FL United States 2018;33 (Supplement 1):S21-S22.

(81) Orozco JL, Valderrama-Chaparro JA, Pinilla-Monsalve GD, Molina-Echeverry MI, Perez Castano AM, riza-Araujo Y, Prada SI, Takeuchi Y. Parkinson's disease prevalence, age distribution and staging in Colombia. Neurol Int 2020 Jul 10;12(1):8401.

(82) Fereshtehnejad S-M, Shafieisabet M, Rahmani A, Delbari A, Lokk J. Cardiovascular and cerebrovascular disorders are associated with a higher prevalence of Parkinsonism: Data from a neuroepidemiologic community-based survey. Movement Disorders Conference: 2020;(Supplement 2):June.

(83) Deischinger C, Dervic E, Kaleta M, Klimek P, Kautzky-Willer A. Diabetes Mellitus is Associated with a Higher Relative Risk for Parkinson's Disease in Women than in Men. J Parkinsons Dis 2021;11(2):793-800.

(84) Abukhalil F, Djenderedjian R, Mehta B, Saito EK, Diaz N, Chung J, McMurtray AM. Diabetes mellitus type two and decreased relative risk for Parkinson's Disease: A community based cross-sectional study. Journal of Parkinson's Disease Conference: 4th World Parkinson Congress, WPC 2016;(Supplement 1):2016.

(85) Wahlqvist ML, Lee MS, Hsu CC, Chuang SY, Lee JT, Tsai HN. Metformin-inclusive sulfonylurea therapy reduces the risk of Parkinson's disease occurring with Type 2 diabetes in a Taiwanese population cohort. Parkinsonism Relat Disord 2012 Jul;18(6):753-8.

(86) Sun Y, Chang YH, Chen HF, Su YH, Su HF, Li CY. Risk of Parkinson disease onset in patients with diabetes: a 9-year population-based cohort study with age and sex stratifications. Diabetes Care 2012 May;35(5):1047-9.

(87) Lai S-W, Liao K-F, Lin C-L, Sung F-C. Irritable bowel syndrome correlates with increased risk of Parkinson's disease in Taiwan. European Journal of Epidemiology 2014;29(1):57-62.

(88) Shen CH, Chou CH, Liu FC, Lin TY, Huang WY, Wang YC, Kao CH. Association Between Tuberculosis and Parkinson Disease: A Nationwide, Population-Based Cohort Study. Medicine (Baltimore) 2016 Feb;95(8):e2883.

(89) Lai S-W, Lin C-L, Liao K-F. Glaucoma correlates with increased risk of Parkinson's disease in the elderly: a national-based cohort study in Taiwan. Current Medical Research and Opinion 2017;33(8):1511-6.

(90) Roh JH, Lee S, Yoon JH. Metabolic Syndrome and Parkinson's Disease Incidence: A Nationwide Study Using Propensity Score Matching. Metab Syndr Relat Disord 2020 Sep 1;19(1):1-7.

(91) Won JH, Byun SJ, Oh BM, Kim HJ, Park SJ, Seo HG. Pneumonia risk and its associated factors in Parkinson's disease: A National Database Study. J Neurol Sci 2020 Aug 15;415:116949.

(92) Chung SJ, Kim MJ, Kim J, Ryu HS, Kim YJ, Kim SY, Lee JH. Association of type 2 diabetes GWAS loci and the risk of Parkinson's and Alzheimer's diseases. Parkinsonism Relat Disord 2015 Dec;21(12):1435-40.

(93) Cereda E, Barichella M, Pedrolli C, Klersy C, Cassani E, Caccialanza R, Pezzoli G. Diabetes and risk of Parkinson's disease: a systematic review and meta-analysis. Diabetes Care 2011 Dec;34(12):2614-23.

(94) Noyce AJ, Bestwick JP, Silveira-Moriyama L, Hawkes CH, Giovannoni G, Lees AJ, Schrag A. Meta-analysis of early nonmotor features and risk factors for Parkinson disease. Ann Neurol 2012 Dec;72(6):893-901.

(95) Mounika P, Venkat A, Chaitanya P, Dev K. Meta-analysis of Parkinson's disease risk with hypertension, serum total cholesterol, and diabetes mellitus. Value in Health Conference: ISPOR 15th Annual European Congress Berlin Germany Conference Publication 2012;15(7):A519-A520.

(96) Lu L, Fu DL, Li HQ, Liu AJ, Li JH, Zheng GQ. Diabetes and risk of Parkinson's disease: an updated meta-analysis of case-control studies. PLoS One 2014;9(1):e85781.

(97) Yue X, Li H, Yan H, Zhang P, Chang L, Li T. Risk of Parkinson Disease in Diabetes Mellitus: An Updated Meta-Analysis of Population-Based Cohort Studies. Medicine (Baltimore) 2016 May;95(18):e3549.

(98) Komici K, Femminella GD, Bencivenga L, Rengo G, Pagano G. Diabetes Mellitus and Parkinson's Disease: A Systematic Review and Meta-Analyses. J Parkinsons Dis 2021;11(4):1585-96.

(99) Liu W, Tang J. Association between diabetes mellitus and risk of Parkinson's disease: A prisma-compliant meta-analysis. Brain Behav 2021 Aug;11(8):e02082.

(100) Chohan H, Senkevich K, Patel RK, Bestwick JP, Jacobs BM, Bandres CS, Gan-Or Z, Noyce AJ. Type 2 Diabetes as a Determinant of Parkinson's Disease Risk and Progression. Mov Disord 2021 Jun;36(6):1420-9.

(101) Pedersen HE, Sandvik CH, Subhi Y, Grauslund J, Pedersen FN. Relationship between Diabetic Retinopathy and Systemic Neurodegenerative Diseases: A Systematic Review and Meta-analysis. Ophthalmology Retina 2022 Feb 1;6(2):139-52.

(102) Chahine LM, Dos SC, Fullard M, Scordia C, Weintraub D, Erus G, Rosenthal L, Davatzikos C, McMillan CT. Modifiable vascular risk factors, white matter disease and cognition in early Parkinson's disease. European Journal of Neurology 2019;26(2):246-e18.

(103) Molsberry S, Bjornevik K, Hughes KC, Zhang ZJ, Jeanfavre S, Clish C, Healy B, Schwarzschild M, Ascherio A. Plasma Metabolomic Markers of Insulin Resistance and Diabetes and Rate of Incident Parkinson's Disease. J Parkinsons Dis 2020;10(3):1011-21.

(104) Castillo-Torres S, Cerda-Contreras C, Martinez-Roque D, Soto-Rincon C, Chavez-Luevanos B, Estrada-Bellman I. Clinical correlates of high cardiovascular risk in parkinson's disease: An observational study. Neurology Conference: 72nd Annual Meeting of the American Academy of Neurology, AAN 2020;(15 Supplement).

(105) Chalmanov V, Vurbanova M. Diabetes mellitus in parkinsonism patients. [Bulgarian]. Vutreshni bolesti 1987;26(1):68-73.

(106) Cosentino M, Martignoni E, Michielotto D, Calandrella D, Riboldazzi G, Pacchetti C, Frigo G, Nappi G, Lecchini S. Medical healthcare use in Parkinson's disease: Survey in a cohort of ambulatory patients in Italy. BMC Health Services Research 2005;5:26.

(107) Becker C, Brobert GP, Johansson S, Jick SS, Meier CR. Diabetes in patients with idiopathic Parkinson's disease. Diabetes Care 2008 Sep;31(9):1808-12.

(108) Trail M, Petersen NJ, Lai EC. Occurrence of diabetes and hypertension in overweight vs. normal weight veterans with Parkinson's disease. Movement Disorders Conference: 14th International Congress of Parkinson's Disease and Movement Disorders Buenos Aires Argentina Conference Publication 2010;25 (SUPPL. 2):S431.

(109) Akushevich I, Kravchenko J, Ukraintseva S, Arbeev K, Kulminski A, Yashin AI. Morbidity risks among older adults with pre-existing age-related diseases. Experimental Gerontology 2013;48(12):1395-401.

(110) Bhattacharjee S, Sambamoorthi U. Diabetes care among elderly medicare beneficiaries with Parkinson's disease and diabetes. Journal of Diabetes and Metabolic Disorders 2015;14(1):75.

(111) Xu K, Alnaji N, Bertoni J, Bhatti D, Chen L-W, Qu M. Comorbid conditions associated with Parkinson's disease: A population-based study from the Nebraska Parkinson's disease registry. Parkinsonism and Related Disorders Conference: 22nd World Congress of the International Association of Parkinsonism and Related Disorders Vietnam 2018;46(Supplement 2):e15-e16.

(112) Heinzel S, Berg D, Gasser T, Chen H, Yao C, Postuma RB. Update of the MDS research criteria for prodromal Parkinson's disease. Movement Disorders 2019;34(10):1464-70.

(113) Choi HG, Lim J-S, Lee YK, Sim S, Kim M. Mortality and cause of death in South Korean patients with Parkinson's disease: A longitudinal follow-up study using a national sample cohort. BMJ Open 2019;9(9):e029776.

(114) Peretz C, Chillag-Talmor O, Linn S, Gurevich T, El-Ad B, Silverman B, Friedman N, Giladi N. The reduced risk of incident diabetes mellitus in a large population based cohort of Parkinson's disease patients. Movement Disorders Conference: 18th International Congress of Parkinson's Disease and Movement Disorders Stockholm Sweden Conference Publication 2014;29(SUPPL. 1):S548.

(115) Hu G, Antikainen R, Jousilahti P, Kivipelto M, Tuomilehto J. Total cholesterol and the risk of Parkinson disease. Neurology 1972;70(21):20.

(116) Nilsson FM, Kessing LV, Bolwig TG. Increased risk of developing Parkinson's disease for patients with major affective disorder: A register study. Acta Psychiatrica Scandinavica 2001;104(5):380-6.

(117) Wolozin B, Wang SW, Li N-C, Lee A, Lee TA, Kazis LE. Simvastatin is associated with a reduced incidence of dementia and Parkinson's disease. BMC Medicine 2007;5:20.

(118) Hu G, Bidel S, Jousilahti P, Antikainen R, Tuomilehto J. Coffee and tea consumption and the risk of Parkinson's disease. Movement Disorders 2007;22(15):2242-8.

(119) Logroscino G, Gao X, Chen H, Wing A, Ascherio A. Dietary iron intake and risk of Parkinson's disease. American Journal of Epidemiology 2008;168(12):1381-8.

(120) Qiu C, Hu G, Kivipelto M, Laatikainen T, Antikainen R, Fratiglioni L, Jousilahti P, Tuomilehto J. Association of blood pressure and hypertension with the risk of parkinson disease: The national FINRISK study. Hypertension 2011;57(6):1094-100.

(121) Cereda E, Cassani E, Barichella M, Spadafranca A, Caccialanza R, Bertoli S, Battezzati A, Pezzoli G. Low cardiometabolic risk in Parkinson's disease is independent of nutritional status, body composition and fat distribution. Clin Nutr 2012 Oct;31(5):699-704.

(122) Ton TGN, Jain S, Biggs ML, Thacker EL, Strotmeyer ES, Boudreau R, Newman AB, Longstreth J, Checkoway H. Markers of inflammation in prevalent and incident Parkinson's disease in the Cardiovascular Health Study. Parkinsonism and Related Disorders 2012;18(3):274-8.

(123) Jain S, Ton TG, Perera S, Zheng Y, Stein PK, Thacker E, Strotmeyer ES, Newman AB, Longstreth WT. Cardiovascular physiology in premotor Parkinson's disease: A neuroepidemiologic study. Movement Disorders 2012;27(8):988-95.

(124) Lysen TS, Darweesh SKL, Kamran IM, Luik AI, Arfan IM. Sleep and risk of parkinsonism and Parkinson's disease: A population-based study. Brain 2013;142(7):2013-22.

(125) Savica R, Grossardt BR, Bower JH, Ahlskog JE, Rocca WA. Risk factors for Parkinson's disease may differ in men and women: An exploratory study. Hormones and Behavior 2013;63(2):308-14.

(126) Wang I-K, Lin C-L, Wu Y-Y, Chou C-Y, Lin S-Y, Liu J-H, Yen T-H, Huang C-C, Sung F-C. Increased risk of Parkinson's disease in patients with end-stage renal disease: A retrospective cohort study. Neuroepidemiology 2014;42(4):204-10.

(127) Chung S-D, Ho J-D, Hu C-C, Lin H-C, Sheu J-J. Increased risk of parkinson disease following a diagnosis of neovascular age-related macular degeneration: A retrospective cohort study. American Journal of Ophthalmology 2014;157(2):464-9.e1.

(128) Brauer R, Bhaskaran K, Chaturvedi N, Dexter D, Smeeth L, Douglas I. Glitazone antidiabetics and the risk of parkinson's disease. Pharmacoepidemiology and Drug Safety Conference: 30th International Conference on Pharmacoepidemiology and Therapeutic Risk Management Taipei Taiwan (Republic of China) Conference Publication 2014;23 (SUPPL. 1):336.

(129) Saaksjarvi K, Knekt P, Mannisto S, Lyytinen J, Heliovaara M. Prospective study on the components of metabolic syndrome and the incidence of Parkinson's disease. Parkinsonism Relat Disord 2015 Oct;21(10):1148-55.

(130) Connolly JG, Bykov K, Gagne JJ. Thiazolidinediones and Parkinson Disease: A Cohort Study. Am J Epidemiol 2015 Dec 1;182(11):936-44.

(131) Kmietowicz Z. Glitazones are associated with reduced risk of Parkinson's disease. BMJ 2015 Jul 22;351:h3949.

(132) Brauer R, Bhaskaran K, Chaturvedi N, Dexter DT, Smeeth L, Douglas I. Glitazone Treatment and Incidence of Parkinson's Disease among People with Diabetes: A Retrospective Cohort Study. PLoS Med 2015 Jul;12(7):e1001854.

(133) Chen H, Ding D, Wang J, Zhao Q, Meng H, Li H, Gao Y-T, Shu X-O, Tanner CM, Hong Z, Yang G. Parkinson's disease research in a prospective cohort in China. Parkinsonism and Related Disorders 2015;21(10):1200-4.

(134) Li C-H, Chen W-C, Liao W-C, Tu C-Y, Lin C-L, Sung F-C, Chen C-H, Hsu W-H. The association between chronic obstructive pulmonary disease and Parkinson's disease: A nationwide population-based retrospective cohort study. QJM 2015;108(1):39-45.

(135) Connolly JG, Bykov K, Gagne JJ. Thiazolidinediones and parkinson's disease: A cohort study. Pharmacoepidemiology and Drug Safety Conference: 31st International Conference on Pharmacoepidemiology and Therapeutic Risk Management Boston, MA United States Conference Publication 2015;24 (SUPPL. 1):305.

(136) Svenningsson P, Wirdefeldt K, Yin L, Fang F, Markaki I, Efendic S, Ludvigsson JF. Reduced incidence of Parkinson's disease after dipeptidyl peptidase-4 inhibitors-A nationwide case-control study. Mov Disord 2016 Sep;31(9):1422-3.

(137) Svensson E, Horvath-Puho E, Stokholm MG, Sorensen HT, Henderson VW, Borghammer P. Appendectomy and risk of Parkinson's disease: A nationwide cohort study with more than 10 years of follow-up. Movement Disorders 2016;31(12):1918-22.

(138) Tsai H-H, Liou H-H, Muo C-H, Lee C-Z, Yen R-F, Kao C-H. Hepatitis C virus infection as a risk factor for Parkinson disease. Neurology 2016;86(9):840-6.

(139) Wang H-I, Ho Y-C, Huang Y-P, Pan S-L. Migraine is related to an increased risk of Parkinson's disease: A population-based, propensity score-matched, longitudinal follow-up study. Cephalalgia 2016;36(14):1316-23.

(140) Shi Q, Liu S, Fonseca V, Shi L. The effect of metformin exposure on neurodegenerative disease among elder adult veterans with diabetes mellitus. Diabetes Conference: 76th Scientific Sessions of the American Diabetes Association, ADA 2016;(Supplement 1):2016.

(141) Brakedal B, Flones I, Reiter SF, Torkildsen O, Dolle C, Assmus J, Haugarvoll K, Tzoulis C. Glitazone use associated with reduced risk of Parkinson's disease. Mov Disord 2017 Nov;32(11):1594-9.

(142) Kuan YC, Huang KW, Lin CL, Hu CJ, Kao CH. Effects of metformin exposure on neurodegenerative diseases in elderly patients with type 2 diabetes mellitus. Prog Neuropsychopharmacol Biol Psychiatry 2017 Oct 3;79(Pt B):77-83.

(143) Cheng KKW, Swallow DMA, Grosset KA, Grosset DG. Statin usage, vascular diagnosis and vascular risk factors in Parkinson's disease. Scottish Medical Journal 2017;62(3):104-9.

(144) Chen H, Kwong JC, Copes R, Tu K, Villeneuve PJ, Van DA, Hystad P, Martin RV, Murray BJ, Jessiman B, Wilton AS, Kopp A, et al. Living near major roads and the incidence of dementia, Parkinson's disease, and multiple sclerosis: a population-based cohort study. The Lancet 2017;389(10070):718-26.

(145) Kuan YC, Huang KW. The effect of metformin exposure on neurodegenerative diseases (dementia and Parkinson's disease) in elder patients with diabetes mellitus. Neurodegenerative Diseases Conference: 13th International Conference on Alzheimer's and Parkinson's Diseases, AD/PD 2017;(Supplement 1):2017.

(146) Wu HF, Kao LT, Shih JH, Kao HH, Chou YC, Li IH, Kao S. Pioglitazone use and Parkinson's disease: a retrospective cohort study in Taiwan. BMJ Open 2018 Aug 29;8(8):e023302.

(147) Lin HL, Lin HC, Tseng YF, Chao JC, Hsu CY. Association of thiazolidinedione with a lower risk of Parkinson's disease in a population with newly-diagnosed diabetes mellitus. Ann Med 2018 Aug;50(5):430-6.

(148) Nam GE, Kim SM, Han K, Kim NH, Chung HS, Kim JW, Han B, Cho SJ, Yu JH, Park YG, Choi KM. Metabolic syndrome and risk of Parkinson disease: A nationwide cohort study. PLoS Medicine 2018;15(8):e1002640.

(149) Li Q, Wang C, Tang H, Chen S, Ma J. Stroke and Coronary Artery Disease Are Associated with Parkinson's Disease. Canadian Journal of Neurological Sciences 2018;45(5):559-65.

(150) Shin S, Burnett RT, Kwong JC, Hystad P, Van DA, Brook JR, Copes R, Tu K, Goldberg MS, Villeneuve PJ, Martin RV, Murray BJ, et al. Effects of ambient air pollution on incident Parkinson's disease in Ontario, 2001 to 2013: A population-based cohort study. International Journal of Epidemiology 2018;47(6):2038-48.

(151) Zhu Y, Pu J, Chen Y, Zhang B. Decreased risk of Parkinson's disease in diabetic patients with thiazolidinediones therapy: An exploratory meta-analysis. PLoS One 2019;14(10):e0224236.

(152) Shi Q, Liu S, Fonseca VA, Thethi TK, Shi L. Effect of metformin on neurodegenerative disease among elderly adult US veterans with type 2 diabetes mellitus. BMJ Open 2019 Jul 30;9(7):e024954.

(153) Jeong S-M, Jang W, Shin DW. Association of statin use with Parkinson's disease: Dose-response relationship. Movement Disorders 2019;34(7):1014-21.

(154) Wolfson C, Fereshtehnejad S-M, Pasquet R, Postuma R, Keezer MR. High burden of neurological disease in the older general population: results from the Canadian Longitudinal Study on Aging. European Journal of Neurology 2019;26(2):356-62.

(155) Jeong SM, Han K, Kim D, Rhee SY, Jang W, Shin DW. Body mass index, diabetes, and the risk of Parkinson's disease. Mov Disord 2020 Feb;35(2):236-44.

(156) Brauer R, Wei L, Ma T, Athauda D, Girges C, Vijiaratnam N, Auld G, Whittlesea C, Wong I, Foltynie T. Diabetes medications and risk of Parkinson's disease: a cohort study of patients with diabetes. Brain 2020 Oct 4;143(10):3067-76.

(157) Chung HS, Lee JS, Kim JA, Roh E, Lee YB, Hong SH, Yu JH, Kim NH, Yoo HJ, Seo JA, Kim SG, Kim NH, et al. Fasting plasma glucose variability in midlife and risk of Parkinson's disease: A nationwide population-based study. Diabetes Metab 2020 Oct 8;47(3):101195.

(158) Yahalom G, Rigbi A, Israeli-Korn S, Krohn L, Rudakou U, Ruskey JA, Benshimol L, Tsafnat T, Gan-Or Z, Hassin-Baer S, Greenbaum L. Age at Onset of Parkinson's Disease among Ashkenazi Jewish Patients: Contribution of Environmental Factors, LRRK2 p.G2019S and GBA p.N370S Mutations. Journal of Parkinson's Disease 2020;10(3):1123-32.

(159) Kim IY, Yang TO, Heath AK, Simpson RF, Reeves GK, Green J, Floud S, Brown A, Hunter DJ, Beral V, Sweetland S. Alcohol intake and Parkinson's disease risk in the million women study. Movement Disorders 2020;35(3):443-9.

(160) Choi S, Jahng WJ, Park SM, Jee D. Association of Age-Related Macular Degeneration on Alzheimer or Parkinson Disease: A Retrospective Cohort Study. American Journal of Ophthalmology 2020;210:41-7.

(161) Brauer R, Wei L, Ma T, Athauda D, Girges C, Vijiaratnam N, Auld G, Whittlesea C, Wong I, Foltynie T. Differential risk of Parkinsons disease according to diabetes treatments: A cohort study. Pharmacoepidemiology and Drug Safety Conference: 36th International Conference on Pharmacoepidemiology and Therapeutic Risk Management Virtual 2020;29 (SUPPL 3):58-9.

(162) Hussain S, Singh A, Baxi H, Taylor B, Burgess J, Antony B. Thiazolidinedione use is associated with reduced risk of Parkinson's disease in patients with diabetes. Movement Disorders Conference: MDS International Congress Virtual 2020;35 (SUPPL 1):S77.

(163) Hussain S, Singh A, Baxi H, Taylor B, Burgess J, Antony B. Thiazolidinedione use is associated with reduced risk of Parkinson's disease in patients with diabetes: a meta-analysis of real-world evidence. Neurological Sciences 2020;41(12):3697-703.

(164) Melendez-Flores JD, Millan-Alanis JM, Gonzalez-Martinez A, varez-Villalobos NA, Estrada-Bellmann I. Does glitazone treatment have a role on the prevention of Parkinson's disease in adult diabetic population? A systematic review. Metabolic Brain Disease 2020;35(7):1067-75.

(165) Sandulescu MC, Vanenkevort E, Gupta M, Zand R. Male gender and the risk of parkinson's disease in an essential tremor population. Movement Disorders Clinical Practice Conference: 3rd Pan American Parkinson's Disease and Movement Disorders Congress Miami, FL United States 2020;7 (Supplement 1):S57.

(166) Chang YH, Yen SJ, Chang YH, Wu WJ, Lin KD. Pioglitazone and statins lower incidence of Parkinson disease in patients with diabetes mellitus. Eur J Neurol 2021 Sep 23;28(2):430-7.

(167) Yen S-J, Chang Y-H, Wu W-J, Lin K-D. Pioglitazone and statins lower incidence of Parkinson disease in patients with diabetes mellitus. European Journal of Neurology 2021;28(2):430-7.

(168) Spiers NA, Matthews RJ, Jagger C, Matthews FE, Boult C, Robinson TG, Brayne C. Diseases and impairments as risk factors for onset of disability in the older population in England and Wales: Findings from the medical research council cognitive function and ageing study. Journals of Gerontology - Series A Biological Sciences and Medical Sciences 2005;60(2):248-54.

(169) Wu Y-H, Lee W-J, Chen Y-H, Chang M-H, Lin C-H. Premotor symptoms as predictors of outcome in parkinsons disease: A case-control study. PLoS ONE 2016;11(8):e0161271.

(170) Xu Y, Yang J, Shang H. Meta-analysis of risk factors for Parkinson's disease dementia. Translational Neurodegeneration 2016;5(1):11.

(171) Secnik J, Cermakova P, Fereshtehnejad SM, Dannberg P, Johnell K, Fastbom J, Winblad B, Eriksdotter M, Religa D. Diabetes in a Large Dementia Cohort: Clinical Characteristics and Treatment From the Swedish Dementia Registry. Diabetes Care 2017 Sep;40(9):1159-66.

(172) Pagano G, Polychronis S, Wilson H, Giordano B, Ferrara N, Niccolini F, Politis M. Diabetes mellitus and Parkinson disease. Neurology 2018;90(19):E1654-E1662.

(173) Mirzae M, Abbasi MH, Shahidi GA, Hosseinifakhr Q, Sina F, Habibi SA. Diabetes increases the incidence of levodopa-induced dyskinesia in parkinson's disease; a case-control study. Journal of Advances in Medical and Biomedical Research 2021;29(136):257-62.

(174) Davis S. Strong link found between type 2 diabetes and Parkinson's disease. Geriatrics 2007;62(5):25.

(175) Alsomali N, Mujtaba A, AlSamrani A, Marar S, Mohammad T. Relationship of metabolic markers in Parkinson's disease. Movement Disorders Conference: 21st International Congress of Parkinson's Disease and Movement Disorders Vancouver, BC Canada 2017;32 (Supplement 2):362.

(176) Zimering MB, Patel D, Bahn G. Type 2 Diabetes Predicts Increased Risk of Neurodegenerative Complications in Veterans Suffering Traumatic Brain Injury. J Endocrinol Diabetes 2019;6(3):137.

(177) Peng Z, Zhou R, Liu D, Cui M, Yu K, Yang H, Li L, Liu J, Chen Y, Hong W, Huang J, Wang C, et al. Association Between Metabolic Syndrome and Mild Parkinsonian Signs Progression in the Elderly. Front Aging Neurosci 2021;13:722836.

(178) Markaki I, Ntetsika T, Sorjonen K, Svenningsson P. Euglycemia Indicates Favorable Motor Outcome in Parkinson's Disease. Mov Disord 2021 Jun;36(6):1430-4.

(179) Ou R, Wei Q, Hou Y, Zhang L, Liu K, Lin J, Jiang Z, Song W, Cao B, Shang H. Effect of diabetes control status on the progression of Parkinson's disease: A prospective study. Ann Clin Transl Neurol 2021 Apr;8(4):887-97.

(180) Li HJ, Yu Y, Chen Y, Liang HY. Vascular risk factors aggravate the progression of Parkinson's disease: a five-year follow-up study in Chinese patients. Int J Clin Exp Med 2015;8(6):9897-903.

(181) Pagano G, Polychronis S, Wilson H, Giordano B, Ferrara N, Niccolini F, Politis M. Diabetes mellitus and Parkinson disease. Neurology 2018 May 8;90(19):e1654-e1662.

(182) Miller-Patterson C, Han J, Yaffe K, Rosso AL, Launer LJ, Kritchevsky SB, Boudreau RM, Rosano C. Clinical and neuroimaging correlates of progression of mild parkinsonian signs in community-dwelling older adults. Parkinsonism Relat Disord 2020 Jun;75:85-90.

(183) Pagano G, Polychronis S, Wilson H, Niccolini F, Politis M. Diabetes mellitus and Parkinson's disease. European Journal of Neurology Conference: 3rd Congress of the European Academy of Neurology Netherlands 2017;24(Supplement 1):566.

(184) Pagano G, Polychronis S, Wilson H, Niccolini F, Politis M. Diabetes mellitus and Parkinson's disease. Movement Disorders Conference: 21st International Congress of Parkinson's Disease and Movement Disorders Canada 2017;32(Supplement 2):80.

(185) Elbaz A, Moisan F. Update in the epidemiology of Parkinson's disease. Curr Opin Neurol 2008 Aug;21(4):454-60.

(186) Chen S-Y, Tsai S-T. The epidemiology of Parkinson's disease. Tzu Chi Medical Journal 2010;22(2):73-81.

(187) Wirdefeldt K, Adami H-O, Cole P, Trichopoulos D, Mandel J. Epidemiology and etiology of Parkinson's disease: A review of the evidence. European Journal of Epidemiology 2011;26(SUPPL. 1):S1-S58.

(188) viles-Olmos I, Limousin P, Lees A, Foltynie T. Parkinson's disease, insulin resistance and novel agents of neuroprotection. Brain 2013 Feb;136(Pt 2):374-84.

(189) Santiago JA, Potashkin JA. System-based approaches to decode the molecular links in Parkinson's disease and diabetes. Neurobiology of Disease 2014;72(Part A):84-91.

(190) Zhang P, Tian B. Metabolic syndrome: an important risk factor for Parkinson's disease. Oxid Med Cell Longev 2014;2014:729194.

(191) Bellou V, Belbasis L, Tzoulaki I, Evangelou E, Ioannidis JP. Environmental risk factors and Parkinson's disease: An umbrella review of meta-analyses. Parkinsonism Relat Disord 2016 Feb;23:1-9.

(192) Ascherio A, Schwarzschild MA. The epidemiology of Parkinson's disease: risk factors and prevention. The Lancet Neurology 2016;15(12):1257-72.

(193) Delamarre A, Meissner WG. Epidemiology, environmental risk factors and genetics of Parkinson's disease. Presse Medicale Part 1 2017;46(2):175-81.

(194) Mule NK, Singh JN. Diabetes Mellitus to Neurodegenerative Disorders: Is Oxidative Stress Fueling the Flame? CNS Neurol Disord Drug Targets 2018;17(9):644-53.

(195) Biosa A, Outeiro TF, Bubacco L, Bisaglia M. Diabetes Mellitus as a Risk Factor for Parkinson's Disease: a Molecular Point of View. Mol Neurobiol 2018 Nov;55(11):8754-63.

(196) Sergi D, Renaud J, Simola N, Martinoli M-G. Diabetes, a contemporary risk for Parkinson's disease: Epidemiological and cellular evidences. Frontiers in Aging Neuroscience 2019;11:302.

(197) Hassan A, Sharma KR, Mishra R, Gautam J, Alaref A, Jahan N. Diabetes Mellitus and Parkinson's Disease: Shared Pathophysiological Links and Possible Therapeutic Implications. Cureus 2020 Aug 18;12(8):e9853.

(198) Cheong JLY, de Pablo-Fernandez E, Foltynie T, Noyce AJ. The Association Between Type 2 Diabetes Mellitus and Parkinson's Disease. J Parkinsons Dis 2020;10(3):775-89.

(199) Potashkin J, Huang X, Becker C, Chen H, Foltynie T, Marras C. Understanding the links between cardiovascular disease and Parkinson's disease. Mov Disord 2020 Jan;35(1):55-74.

(200) Chen Y, Sun X, Lin Y, Zhang Z, Gao Y, Wu IXY. Non-Genetic Risk Factors for Parkinson's Disease: An Overview of 46 Systematic Reviews. J Parkinsons Dis 2021;11(3):919-35.

(201) Gomez-Chavarin M, Morales-Gomez M. Metabolic sindrome and Parkinson's disease. [Spanish]. Archivos de Neurociencias 2021;25(2):34-44.

Supplementary Table 2. World Cancer Research Fund grading criteria

Grading	Criteria
Convincing	A convincing relationship should be robust enough to be highly unlikely to be modified in the foreseeable future as new evidence accumulates. All of the following are generally required:<br><br>- Evidence from more than one study type<br><br>- Evidence from at least two independent cohort studies<br><br>- No substantial unexplained heterogeneity within or between study types or in different populations relating to the presence or absence of an association, or direction of effect<br><br>- Good quality studies to exclude with confidence the possibility that the observed association results from random or systematic error, including confounding, measurement error, and selection bias<br><br>- Presence of a plausible biological gradient in the association. Such a gradient need not be linear or even in the same direction across different levels of exposure, so long as this can be explained plausibly<br><br>- Strong and plausible experimental evidence, either from human studies or relevant animal models, that typical human exposures can lead to relevant outcomes
Probable	All of the following are generally required:<br><br>- Evidence from at least two independent cohort studies, or at least five case-control studies<br><br>- No substantial unexplained heterogeneity within or between study types or in different populations relating to the presence or absence of an association, or direction of effect<br><br>- Good quality studies to exclude with confidence the possibility that the observed association results from random or systematic error, including confounding, measurement error, and selection bias<br><br>- Evidence for biological plausibility
Limited - suggestive	All of the following are generally required:<br><br>- Evidence from at least two independent cohort studies, or at least five case-control studies<br><br>- The direction of effect is generally consistent though some unexplained heterogeneity may be present

	- Evidence for biological plausibility
Limited - no conclusion	Evidence is so limited that no firm conclusion can be made, but this does not mean that there is evidence of no relationship. The evidence might be graded "limited - no conclusion" for several reasons:<br><br>- limited number of studies<br><br>- inconsistency of direction of effect<br><br>- poor quality of studies (e.g. lack of adjustment for known confounders)<br><br>- or any combination of these factors
Substantial effect on risk unlikely	All of the following are generally required:<br><br>- Evidence from more than one study type<br><br>- Evidence from at least two independent cohort studies<br><br>- Summary estimate of effect close to 1.0 for comparison of high versus low exposure categories<br><br>- No substantial unexplained heterogeneity within or between study types or in different populations<br><br>- Good quality studies to exclude with confidence the possibility that the absence of association results from random or systematic error, including inadequate power, imprecision or error in exposure measurement, inadequate range of exposure, confounding, and selection bias<br><br>- Absence of a demonstrable biological gradient (dose response)<br><br>- Absence of strong and plausible experimental evidence, either from human studies or relevant animal models, that typical human exposures lead to relevant outcomes

Specific upgrading factors:
1) Presence of a plausible biological gradient (dose response) in the association. Such a gradient need not be linear or even in the same direction across the different levels of exposure, so long as this can be explained plausibly.
2) A particularly large summary effect size (an odds ratio or relative risk of 2.0 or more, depending on the unit of exposure) after appropriate control for confounders.
3) Evidence from randomised trials in humans.
4) Evidence from appropriately controlled experiments demonstrating one or more plausible and specific mechanisms actually operating in humans.
5) Robust and reproducible evidence from experimental studies in appropriate animal models showing that typical human exposures can lead to relevant health outcomes.

Supplementary Table 3. Modified Newcastle Ottawa Scale score for the individual studies

Author, year	Selection			Comparability	Outcome assessment			Total
	Selection of non-exposed cohort	Exposure ascertainment	Demonstration of outcome not present at start	0.25 points for each adjustment	Outcome assessment	Long enough follow-up	Adequacy of follow-up	
Simon, 2007	1	1	1	0.75	1	1	0	5.75
Hu, 2007	1	1	1	2	1	1	0	7
Driver, 2008	1	0	1	1.75	1	1	0	5.75
Xu, 2011	1	0	1	2	1	1	0	6
Palacios, 2011	1	0	1	2	1	1	0	6
Pupillo, 2016	1	1	1	2	0.5	0	0	5.5
Yang, 2017	1	1	1	2	0.5	1	1	7.5
Lee, 2018	1	1	1	2	0.5	1	0	6.5
De Pablo-Fernandez, 2018	1	1	1	1.25	0.5	1	0	5.75
Kummer, 2019	1	1	1	1	0.5	1	0	5.5
Kizza, 2019	1	0	0	2	0.5	1	0	4.5
Azizova, 2019	1	1	0	0.5	0.5	1	1	5
Jacobs, 2020	1	1	1	1	0.5	1	0	5.5

Sanchez-Gomez, 2021	1	1	1	1.25	1	1	0	6.25
